# Is adiposity associated with back and lower limb pain? A systematic review

**DOI:** 10.1371/journal.pone.0256720

**Published:** 2021-09-14

**Authors:** Waruna L. Peiris, Flavia M. Cicuttini, Sultana Monira Hussain, Mahnuma M. Estee, Lorena Romero, Tom A. Ranger, Jessica L. Fairley, Emily C. McLean, Donna M. Urquhart

**Affiliations:** 1 Department Epidemiology and Preventive Medicine, School of Public Health and Preventive Medicine, Monash University, Melbourne, Victoria, Australia; 2 The Ian Potter Library, The Alfred Hospital, Melbourne, Victoria, Australia; University of Tennessee Health Science Center College of Graduate Health Sciences, UNITED STATES

## Abstract

**Background:**

Back and lower limb pain have a major impact on physical function and quality of life. While obesity is a modifiable risk factor for musculoskeletal pain, the role of adiposity is less clear. This systematic review aimed to examine the relationship between both adiposity and its distribution and back and lower limb pain.

**Methods:**

A systematic search of electronic databases was conducted to identify studies that examined the association between anthropometric and/or direct measures of adiposity and site specific musculoskeletal pain. Risk of bias was assessed and a best evidence synthesis was performed.

**Results:**

A total of 56 studies were identified which examined 4 pain regions, including the lower back (36 studies), hip (two studies), knee (13 studies) and foot (eight studies). 31(55%) studies were assessed as having low to moderate risk of bias. 17(30%) studies were cohort in design. The best evidence synthesis provided evidence of a relationship between central adiposity and low back and knee pain, but not hip or foot pain. There was also evidence of a longitudinal relationship between adiposity and the presence of back, knee and foot pain, as well as incident and increasing foot pain.

**Conclusions:**

This systematic review provides evidence of an association between both body fat and its central distribution and low back and knee pain, and a longitudinal relationship between adiposity and back, knee and foot pain. These results highlight the potential for targeting adiposity in the development of novel treatments at these sites.

## Introduction

Musculoskeletal conditions are a leading disease burden worldwide. They are not only the second most common cause of global disability, but disability-adjusted life years (DALYs) for musculoskeletal conditions have increased alarmingly, with a rise of up to 62% between 1990 and 2016 [1]. One in three people worldwide live with a musculoskeletal condition, which is characterised by pain and disability, leads to reduced quality of life, and results in a huge economic burden [2]. Back and lower limb pain are highly prevalent musculoskeletal conditions and make a major contribution to their increasing burden at an individual, familial and societal level. Current efforts to reduce the profound impact of these conditions have focussed on determining modifiable risk factors for management and prevention.

Obesity is an escalating, global epidemic. The 2016 Global Burden of Disease Study showed that the prevalence of obesity is not only increasing, but obese people are actually living longer, which allows for the development of co-existing conditions, such as musculoskeletal pain [3]. There is growing evidence to indicate that obesity is a modifiable risk factor for musculoskeletal pain at different sites. A meta-analysis by Shiri and colleagues reported overweight and obesity, measured by weight and body mass index (BMI), to be risk factors for low back pain [4], while a systematic review by Butterworth et al. found increased BMI to be strongly associated with non-specific foot pain in the general population [5]. While these reviews provide evidence for a relationship between obesity, measured by body weight or BMI, and musculoskeletal pain, they do not account for body composition and thus don’t consider the individual contributions of fat mass and lean tissue mass (or muscle mass). This is of particular importance given there is evidence to show that fat mass or adiposity and muscle mass have different roles in the pathogenesis of musculoskeletal disease [6,7].

There is growing evidence to show that adiposity plays an important role in musculoskeletal pain. Adipose tissue acts as an endocrine organ, releasing a host of pro-inflammatory cytokines and adipokines [8], which can heighten inflammatory changes leading to destruction of tissue [9] and increasing pain and disability. A single systematic review has examined the relationship between body fat and musculoskeletal pain [10], reporting a positive cross-sectional association between higher body fat and single-site joint pain in the low back, knee and foot. However, no conclusions could be drawn from longitudinal data regarding the role of adiposity in back and lower limb pain, as there was a lack of available high quality, cohort studies. Moreover, the review focussed on studies that used direct measures of body fat, such as fat mass and percentage of body fat, and excluded those that examined anthropometric measures, such as waist circumference and waist hip ratio [11], thus limiting the opportunity to examine role of fat distribution, particularly central adiposity.

Understanding the role of adiposity in musculoskeletal pain, particularly back and lower limb pain, has huge potential to inform the development of novel prevention and treatment approaches, as well as further our understanding of mechanisms underlying the relationship between obesity and musculoskeletal pain. The aims of this systematic review were to: (i) examine the relationship between central adiposity and back and lower limb pain and (ii) investigate the longitudinal association between adiposity and both the presence, incidence and progression of pain at these sites.

## Methods

This systematic review was conducted according to Preferred Reporting Items for Systematic Reviews and Meta-Analyses (PRISMA) statement (see S1 Checklist) [12].

### Data sources and searches

We performed electronic searches of six databases, including MEDLINE, Embase, CINAHL, Cochane Central Register of Controlled Trials, Scopus and Web of Science from database inception to February 2, 2021. Our initial search for studies was conducted using text words and subject terms on three key databases and then based on this, we developed the search strategy, with subject classification systems investigated for each database and expanded our data sources to include all six databases for our final search. The final searches of all six databases, covering the key concepts of adiposity and musculoskeletal pain, were performed using the appropriate specifications for each database. The comprehensive search strategy for OVID Medline is provided (see S1 Medline Database search strategy in S1 Text). The searches were restricted to adult human studies but not limited based on language. To identify grey literature, we searched Google scholar, using key terms such as ‘adiposity’ and ‘musculoskeletal pain’, from 2011 to 14 February 2021, and Scopus, using our Scopus search strategy and selecting for conference proceedings, from inception to 14 February 2021. In addition, reference lists of reviews and key papers were searched to identify relevant literature.

### Inclusion and exclusion criteria

Studies were included if they investigated the relationship between adiposity and low back or lower limb pain, using at least one measure of adiposity and reporting pain as an outcome measure. Studies that examined adiposity using: (i) anthropometric measures, including waist circumference, hip circumference, waist-hip ratio, waist-height ratio, and skin folds, and (ii) direct fat measures, such as fat mass and body fat percentage, using dual-energy X-ray absorptiometry (DXA) and bioelectrical impedance, were included.

For the purposes of this review, waist circumference was defined as a measurement around the trunk at the midpoint between the lower margin of the least palpable rib and the top of the iliac crest [11]. Hip circumference was considered to be a measure around the hips at the maximum posterior extension of the buttocks, while waist-hip ratio and waist-height ratio were calculated by dividing waist circumference by hip circumference measures, and waist circumference by height, respectively [11]. Skinfold measures assessed the subcutaneous fat thickness and were measured by skinfold calipers [13], while fat mass and body fat percentage were defined as the total mass of adipose tissue or percentage of total adipose tissue of the whole body mass respectively. Central adiposity, an accumulation of both subcutaneous and visceral fat in the lower torso around the abdominal area, was assessed by waist circumference or waist-hip or waist-height ratio measures, which are recommended by the World Health Organization [11].

Data on the presence, incidence and progression of pain in each region was recorded from the included studies where possible. The presence of pain, which was reported from cross-sectional, case-control and cohort studies, was defined as pain recorded at one point in time. For a cohort study, this could have been where adiposity was assessed at baseline and pain was measured at follow-up. Incident pain was defined as where pain was assessed at both baseline and follow-up in a cohort study, with pain absent at baseline and present at follow-up. Moreover, the progression of pain was described where pain was present at both baseline and follow-up in a cohort study and was assessed as increasing, decreasing or not changing over the study period There was no hierarchy given to these pain outcomes, however data from cohort studies were considered the highest level of evidence, followed by case-control studies and then cross-sectional studies.

We excluded studies that: (1) reported BMI or weight only; (2) examined only intramuscular fat; (3) reported pain in the head, neck or upper limb; (4) investigated pain other than musculoskeletal pain, i.e. abdominal pain, cardiac pain; and (5) examined multisite musculoskeletal pain where data specific to the back or lower limb were not reported separately.

### Study selection

Titles and abstracts were assessed by two investigators (WP and TR) for relevance and the full texts were retrieved for relevant studies.

### Data extraction

Data were extracted and tabulated by two reviewers (WP and TR) independently. Studies were categorized based on: (i) the site of pain investigated (low back, hip, knee and foot), (ii) their study design (cross-sectional, case-control or cohort) and (iii) the type of adipose measure reported (anthropometric versus direct fat measures). Data extracted from the studies included (1) author and year of publication, (2) study population characteristics (number of study participants, gender (% women), mean (SD) age, recruitment source), (3) assessment method and measure for adiposity and pain, (4) results (OR/RR, 95%CI) and (5) conclusions.

### Risk of bias assessment

To assess the risk of bias of the included studies, two reviewers (TR and JF) independently assessed the included studies using the Cochrane risk of bias assessment [14]. The Cochrane risk of bias assessment examines the internal and external validity of the included studies, based on four items for cross-sectional studies and five items for cohort studies, with each item scored as low, moderate or high risk of bias. An overall assessment was then given for each study; low if every individual item scored low, moderate if all items scored low except either one high or two moderate, or high if individual items scored more than one high or more than two moderate.

### Best evidence synthesis

A best evidence synthesis was used to summarise the data. It was not possible to perform a meta-analysis as there was substantial clinical and methodological heterogeneity across the studies, including differences in the clinical populations investigated, risk factors and outcomes measured, and statistical data and analyses performed. Based on the study design, the number of studies, the risk of bias rating, and consistency of the results of the studies, levels of evidence for the association between adiposity and pain was determined for each region. The studies were ranked according to their design, with cohort studies considered the highest level of evidence, followed by case-control studies and then cross-sectional studies. Studies were classified as having an association (“positive” or “negative”) if the association reported was statistically significant according to the authors’ predetermined alpha value (or p < .05 where this was not reported) or where the confidence interval for an odds ratio did not cross one.

The levels of evidence used were adapted from the Lievense’s standardized criteria [15], which have been used previously in observational studies of musculoskeletal conditions [16]. They included: evidence of an association, conflicting evidence, limited evidence or no evidence. ‘Evidence of an association’ was defined as consistent findings in multiple, cohort studies, while ‘conflicting evidence’ was defined as inconsistent findings across the number and types of studies. ‘Limited evidence’ was defined as consistent findings in a small number of studies, including a single cohort study or one or two case-control or cross-sectional studies, and ‘no evidence’ was used when there are no studies that provided any evidence.

## Results

### Identification of studies

**Fig 1:**
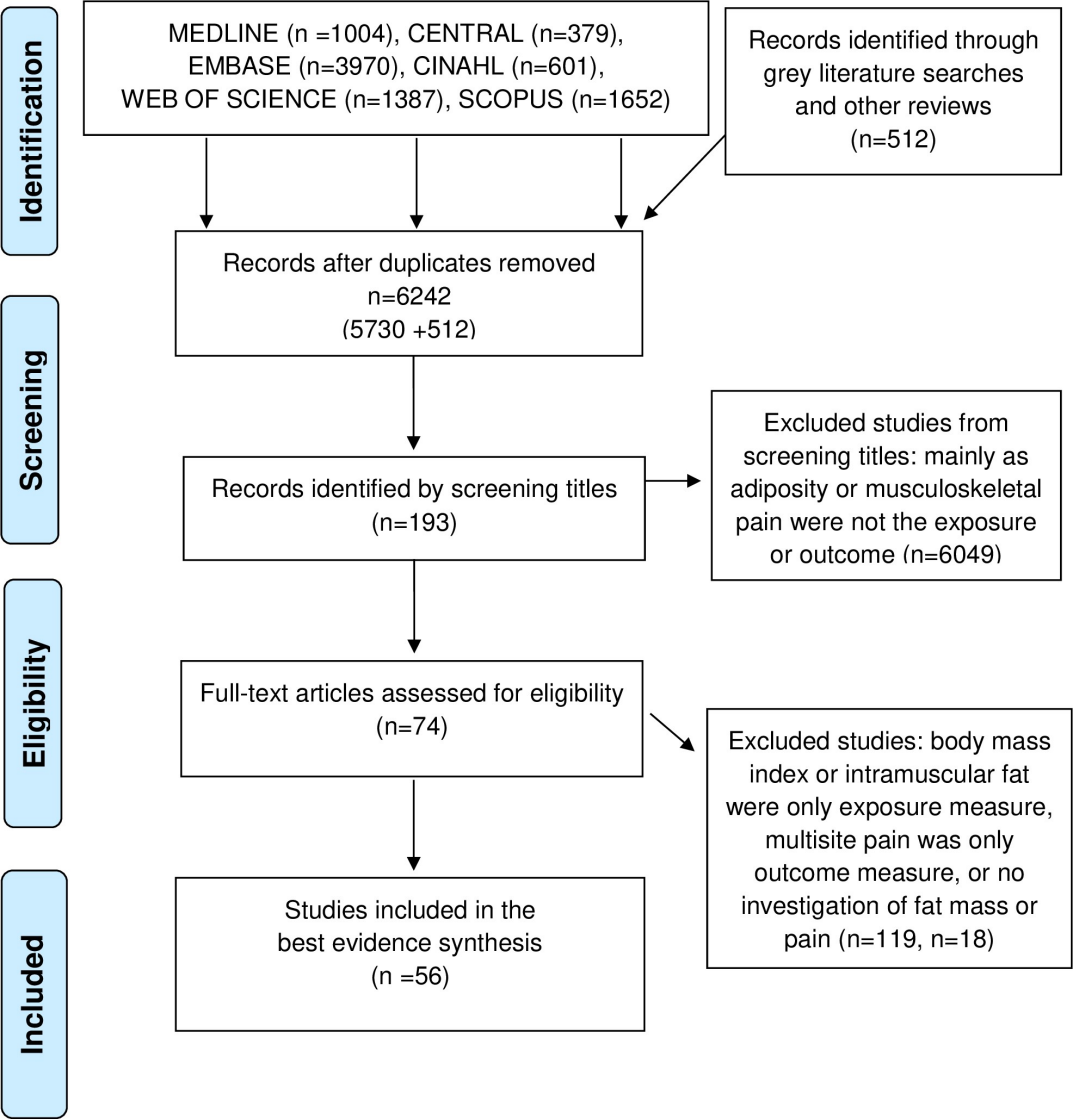
PRISMA flow diagram.

After removal of the duplicates, 6,242 records remained (Fig 1). A total of 6,049 studies were excluded based on the screening of titles and abstracts, leaving 193 studies for full text analysis. A further 74 studies were excluded as they did not meet the review’s inclusion criteria: 37 studies only included BMI as their measure of obesity [17–51], 18 studies did not examine any associations between fat mass and pain [50,52–67], nine studies examined adiposity within a specific muscle [68–75], five studies did not specify a site of pain [76–79], two studies only examined multisite pain [80,81], and three studies examined pain in children [82–84].

### Characteristics of the included studies

A total of 56 studies were included in this review (Table 1). Of the included studies, 17 were cohort [85–101], ten were case-control [102–111] and 29 were cross-sectional [6,7,112–138] studies. Twenty one studies were conducted in Australia [6,7,85,89,90,92–94,97–99,101,102,105,106,117,119,122,126,129,134], seven in Japan [95,107,118,121,131,132,136], five in Finland [87,96,123,124,127], four in the USA [86,91,100,112], two in Brazil [128,130], Turkey [109,120], The Netherlands [113,115], India [114,135], Nigeria [116,133], and China [104,110], and one each in, Korea [125], Slovenia [103], Norway [88], United Kingdom [100], Sweden [111], Mexico [137], Egypt [138] and Greece [108]. Of the 56 studies, 36 examined low back pain [6,7,87,88,92,93,95–97,100,103–105,107,108,111–118,121–123,127,130–138], two examined hip pain [94,102], 13 examined knee pain [86,89,91,94,101,109,110,120,124,125,128,129,132], and eight examined foot pain [85,90,94,98,99,106,119,126].

**Table 1:** Characteristics of studies investigating the relationship between adiposity and back and lower limb pain.

Author (Country, year)	Study population	No. of participants (%women) Age (years): Mean (SD)	Method of measuring adiposity	Measure of adiposity	Measure of pain	Risk of bias rating
**Low back pain**						
Cross-sectional studies						
*Anthropometric fat measurement*						
Yoshimoto (Japan, 2019) [136]	Participants who attended an annual health check-up by the ‘All Japan Labour Welfare Foundation’ were recruited.	45,192 (32.1) 50.5 (7.1)	NA	WC	Single question: “Do you have LBP under treatment including follow-up?”	Moderate
Hussien (Egypt, 2019) [138]	Participants examined and diagnosed by their physician and referred for physical therapy.	132 (38) 33.1 (9.23)	Flexible tape measure	WC HC WHR	VAS	Moderate
Kulandaivelan (2018, India) [135]	Participants recruited from a survey of an urban geographical area.	1503 (54.2) 48.2 (13.1)	Non-elastic inch tape	WC	Pain lasting > 1 day in the past 12 months	Moderate
Brady (Australia, 2018) [33]	Participants from a randomised controlled trial of vitamin D in community-based overweight/obese individuals	62 (37.1) 31.3 (8.5)	NA	WC	Single question “Have you had back pain in the past month?”	Moderate
Machado (Brazil, 2018) [130]	Participants from PAINEL study	268 (70.9) 75.5 (6.1)	Not stated	WC	Interview question “pain in last 6 months that did not disappear for at least 30 consecutive days”	High
Ogwumike (Nigeria, 2016) [133]	Participants were post-menopausal women recruited from government secretariats, schools, and hospitals in the local government area of Oyo State.	310 (100) 41–50 yo: 30.3% 51–60 yo: 64.8% 60–65 yo: 4.8%	NA	WC WHR WHtR	Standardized Nordic Musculoskeletal Questionnaire: prevalence of back pain over 12 months.	Low
Chou (Australia, 2016) [7]	Participants from the Geelong Osteoporosis Study, who were randomly recruited from the electoral roll	820 (100) No LBP: 58.1 (17.1) LBP: 62.9 (14.0)	Tape measure	WHR	Chronic Pain Grade Questionnaire	Moderate
Frilander (Finland 2015) [123]	Register of the Finnish Defence Forces	1385 (0) 40.2	Not stated	WC, WHR	Three questions: “Have you ever had LBP?” “Have you had LBP during the preceding 20 days?” “If you had LBP, did it radiate?”	Moderate
Muramoto (Japan, 2014) [132]	Healthy Japanese volunteers who attended a basic health check-up (Yakumo study in 2011–12)	217 (100) 68.3 (5.0)	Non-stretchable measuring tape	WC HC WHR	VAS	Low
Briggs (United States, 2013) [112]	Participants from the NHANES data base (1999–2004)	14206 (52.2) Not specified	Measuring tape	WC	Two specific questions of back pain in the NHANES questionnaire	High
Ojoawo (Nigeria, 2011) [116]	Patients referred from an orthopaedic clinic diagnosed with LBP and no serious complications	64 (100) 52.33(10.24)	Inelastic flexible tape	WHR, WC, HC	VAS Semantic differential scale	High
Perry (Australia, 2009) [117]	Adolescents from the Western Australian Pregnancy Cohort “Raine” study	1608 (48.7) 14.06 (0.20)	Cloth tape	WC	Questionnaire including 3 back pain questions regarding having back pain: ever, in the last month and longer than 3 months	Moderate
Shiri (Finland, 2008) [127]	Participants of the Cardiovascular Risk in Young Finns Study (1980–2001)	2620 (51.8) 31.2 (5.0)	Tape measure	WC, HC, WHR	Structured interview	High
Toda (Japan, 2000) [118]	Japanese participants with chronic low back pain, with or without positive straight leg raise	330 (62) Cases: 59.7(8.7) Controls: 57.6 (8.7)	Segmental bioelectrical impedance	WHR	Not reported	High
Han (The Netherlands, 1997) [113]	Subjects from the MORGAN project randomly recruited from three towns: Amsterdam, Maastricht and Doetinchem.	12,905 (54.4) 42.9 (10.7)	Tape measure	WC, WHR	Questionnaire	High
*Direct fat measurement*						
Endo (Japan, 2019) [131]	Participants recruited from CoHRE study	1314 (59.4) Female No LBP: 72.5 (6.6) LBP: 73.5 (6.5) Male No LBP: 72.9 (6.6) LBP: 72.8 (6.4)	Bioelectrical impedance analysis	Fat mass	Single question: Do you have low back pain at present?	Moderate
Brady (Australia, 2018) [33]	Participants from a randomised controlled trial of vitamin D in community-based overweight/obese participants	62 (37.1) 31.3 (8.5)	Dual x-ray absorptiometry	Fat mass Body fat %	Single question “Have you had back pain in the past month?”	Moderate
Nava-Bringas (Mexico, 2018) [137]	Patients receiving care in the Spinal Rehabilitation Dept of National Rehabilitation Institute	27 (66.7) 58.6 (6.98)	Bioelectric impedance analysis	Fat mass Body fat %	Numerical scale (0–10)	High
Brooks (Australia, 2016) [122]	Recruited through media advertising and leaflet drops	70 (57) Range: 18–76	Tape measure Bioelectrical impedance analysis	Abdominal to lumbar fat mass ratio	VAS	Low
Chou (Australia, 2016) [7]	Participants from the Geelong Osteoporosis Study, who were randomly recruited from the electoral roll	820 (100) No LBP: 58.1 (17.1) LBP: 62.9 (14.0)	Dual x-ray absorptiometry	Fat mass	Chronic Pain Grade Questionnaire	Moderate
Iizuka (Japan, 2015) [121]	Participants were recruited from an annual medical examination concerning life threatening diseases	273 (65.6) 64.3 (13.2)	Bioelectrical impedance analysis	Total body fat mass	Questionnaire regarding presence of LBP and chronic LBP with aid of VAS	Moderate
Bihari (India, 2011) [114]	All age groups and both sexes from Gurgaon and NOIDA in the National Capital Region	2086 (48.4) Range: 10–70	Bioelectric impedance analysis	Total body fat mass	Structured interview	High
Ojoawo (Nigeria, 2011) [116]	Patients referred from an orthopaedic clinic diagnosed with LBP and no serious complications	64 (100) 52.33(10.24)	Mathematical calculations	Body fat %	VAS Semantic differential scale	High
Urquhart (Australia, 2011) [6]	Participants ranging from normal weight to obese from community weight loss clinics or recruited by local media	135(83.1) 47.4 (9.0)	Dual x-ray absorptiometry	Total body fat mass, upper and lower limb fat mass	Chronic Pain Grade Questionnaire	Moderate
Hodselmans (The Netherlands, 2010) [115]	Outpatients diagnosed with nonspecific chronic low back pain	101 (46) 39.2(9.6)	Skin fold calipers	Body fat %	Patient included if complained of LBP for >3 months	High
Toda (Japan, 2000) [118]	Japanese participants with chronic low back pain, with or without a positive straight leg raise	330 (62) Cases: 59.7(8.7) Controls: 57.6 (8.7)	Segmental bioelectrical impedance	Body fat %	Not reported	High
Case control studies						
*Anthropometric fat measurement*						
Dario (Australia 2016) [105]	Population based Murcia Twin Registry	1128 (100) Cases: 53.59 (7.38) Controls: 53.23 (7.38)	Inelastic tape measure	WC, WHR	Single question “Have you ever had chronic LBP, with chronic defined as greater than 6 months?”	High
Yip (China, 2001) [104]	Recruited from University Family Medical Clinic or from previous population-based study	417 (100) NA	Measuring tape	WC, HC, WHR	Back pain for more than one day	High
Hultman (Sweden, 1993) [111]	Recruited from a metropolitan industrial company and the Karolinska Hospital Dept of Orthopaedic Surgery outpatient clinic.	168 (0) Group 1: 50 (3) Group 2: 50 (3) Group 3: 49 (6)	Skin fold calipers	Skin fold measures at the biceps, triceps, subscapular, and supra iliaca crest sites were used to calculate % fat (volume).	3 groups: Group 1: never had LBP or slight LBP impairment Group 2: had several or at least one episode of LBP (but no LBP for 2 months pre-study) Group 3: ≥3 years of chronic LBP, > 3months of sick leave in the previous year	High
*Direct fat measurement*						
Sakai (Japan, 2017) [107]	Participants recruited from the orthopaedic surgery outpatient department	Cases: 100 (55) Controls: 256 (45) Cases: 74.4 (6.0) Controls: 73.2 (7.6)	Dual x-ray absorptiometry	Fat mass, body fat %	Persistent back pain for 3 months	High
Dario (Australia, 2016) [105]	Population based Murcia Twin Registry	1128 (100) Cases: 53.59 (7.38) Controls: 53.23 (7.38)	Bioelectrical impedance analysis	Body fat %	Single question “Have you ever had chronic LBP, with chronic defined as greater than 6 months”	High
Spyropoulos (Greece, 2008) [108]	Participants selected from previous survey of office workers, who were randomly recruited from 3000 employees from 4 of 18 government offices	60 (100) Cases: 41.7 (7.3) Controls: 42.2 (7.3)	Skin fold calipers	Body fat %	Cases of chronic LBP were considered if pain persisted for a minimum of 15 months.	High
Celan (Slovenia, 2005) [103]	Bus drivers recruited from a municipal transport company	112 (0) 44.2 (5.6)	Lorenz’ constitutional index	Body fat %	Single question about previous LBP, with one follow up question regarding number of episodes if yes (no duration specified)	High
Cohort studies						
*Anthropometric fat measurement*						
Muthuri (UK, 2020) [100]	Participants recruited from the MRC National Survey of Health and Development (British cohort study from midlife to age 69 yo).	3426 (49.7) 36, 43, 53, 60–64, 68–69 years	Not stated	WC	All ages (except 68 yo): single question about whether they had sciatica, lumbago or recurring/ severe backache all or most of the time (ever at ages 36 and 43 and in the previous 12 months at ages 53 and 60–64). Age 68: single question about whether they had experienced any ache or pain in the previous month which had lasted for 1 day or longer.	Moderate
Shiri (Finland, 2019) [96]	Participants from Finnish population based surveys, Health 2000 and Health 2001	1850 (55.0) Over 30	Not stated	WC	Participants asked how many days of back pain they have had in the past 12 months.	Moderate
Dario (Australia, 2017) [93]	Participants recruited from Murcia Twin Registry	1098 (47.3) 53.7 (7)	Inelastic tape measure	WC, WHR	Single question “Have you ever suffered from chronic LBP?”	Moderate
Hussain (Australia, 2017) [92]	Participants recruited from AusDiab study	4986 (55.7)	Metal anthropometric tape	WC	Chronic Pain Grade Questionnaire	Moderate
Heuch (Norway, 2015) [88]	Participants recruited from the Nord-Trondelag Health Study (HUNT)	25329 (55) 30–69	Not stated	WC, WHR	Two questions regarding presence and area of pain	High
Shiri (Finland, 2013) [87]	Participants of the Cardiovascular Risk in Young Finns Study (1980–2007) based on the 2001–2007 follow up	1224(52.5) 31.4(5.0)	Tape measure	WC	Single question “Have you had low back trouble (pain, ache, or unpleasant sensations) during the preceding 12 months?” with follow up questions regarding radiating pain.	High
*Direct fat measurement*						
Muthuri (UK, 2020) [100]	Participants recruited from the MRC National Survey of Health and Development (British cohort study from midlife to age 69 yo).	3426 (49.7) 36, 43, 53, 60–64, 68–69 years	Dual X-ray absorptiometry	Fat mass FMI	60–64 years: single question about whether they had sciatica, lumbago or recurring/severe backache all or most of the time in the previous 12 months. 68 years: single question about whether they had experienced any ache or pain in the previous month which had lasted for 1 day or longer.	Moderate
Brady (Australia, 2019) [97]	Participants from local media and public, private and community health clinics	123 (78) 48.6 (8.5)	Dual X-ray absorptiometry	Fat mass	Chronic Pain Grade Questionnaire	Moderate
Dario (Australia, 2017) [93]	Participants recruited from Murcia Twin Registry	1098 (47.3) 53.7 (7)	Bioelectric impedance analysis	Body fat %	Single question “Have you ever suffered from chronic LBP?”	Moderate
Hashimoto (Japan, 2017) [95]	Participants were employees of companies based in the greater Tokyo metropolitan area	1152 (0) 28.0 (4.6)	Skin fold using subcutaneous fat thickness-measuring device	Body fat %	Presence of LBP was obtained using questionnaire with options (none, in the past, present)	High
Hussain (Australia, 2017) [92]	Participants recruited from AusDiab study	4986 (55.7)	Bioelectric impedance analysis	Body fat % Fat mass	Chronic Pain Grade Questionnaire	Moderate
**Hip pain**						
Case control studies						
*Anthropometric fat measurement*						
Fearon (Australia, 2012) [102]	Recruited from private healthcare providers. Participants either had a gluteal tendon reconstruction, hip osteoarthritis or no hip pain.	102 (100) 62(13.3)	Non stretch tape measure	WC, HC, greater trochanter circumference	Trochanteric pain was used to identify hip pain.	High
Cohort						
*Direct fat measurement*						
Pan (Australia 2017) [94]	Tasmania Older Adult Cohort Study	768 (50) 67.1 (7.3)	Dual x-ray absorptiometry	Fat mass, FMI	Presence of pain (yes/no)	Moderate
**Knee pain**						
Cross-sectional studies						
*Anthropometric fat measurement*						
Lee (Korea, 2016) [125]	Fifth Korean National Health and Nutrition Examination Survey	1664 (67.6) 66.99 (0.33)	Dual x-ray absorptiometry	WC	Presence of pain for 30 days from last 3 months and knee pain intensity measured on a scale of 1–10.	Low
Frilander (Finland, 2016) [124]	Register of the Finnish Defence Forces	1913 (0) No knee pain: 34.6 Knee pain: 37.5	Not stated	WC	Three questions: “Have you ever had LBP?” “Have you had LBP during the preceding 20 days?” “If you had LBP, did it radiate?”	High
Muramoto (Japan, 2014) [132]	Healthy Japanese volunteers who attended a basic health check-up (Yakumo study in 2011–12)	217 (100) 68.3 (5.0)	Non-stretchable measuring tape	WC HC WHR	VAS	Low
*Direct fat measurement*						
Alfieri (Brazil, 2017) [128]	Patients referred to the physical therapy department of a private university in São Paulo	107 (87) 61.8 (10.1)	Bioelectric impedance analysis	Fat mass %	WOMAC	Moderate
Lee (Korea, 2016) [125]	Fifth Korean National Health and Nutrition Examination Survey	1664 (67.6) 66.99 (0.33)	Dual x-ray absorptiometry	Leg to whole body fat mass	Presence of pain for 30 days from last 3 months and knee pain intensity measured on a scale of 1–10.	Low
Ozer Kaya (Turkey, 2014) [120]	Volunteers applying at a sports centre for an exercise consultation	149 (100) 42.6 (4.1)	TANITA Body composition analyser	Body fat %, fat mass	VAS	Moderate
Scott (Australia, 2012) [129]	Study conducted within the Tasmania Older Adult Cohort Study, a population based study	357 (50) Males no knee pain: 63.0 (7.3) Males with knee pain: 62.0 (7.2) Females no knee pain: 62.0 (7.0) Females with knee pain: 61.7 (7.5)	Dual x-ray absorptiometry	Body fat %	Single question: Do you have pain at any of these sites today? with a list to choose from including “any knee pain”	Low
Case-control studies						
*Anthropometric fat measurement*						
Li (China, 2016) [110]	Participants attending 2nd Xiangya Hospital for total knee arthroplasty.	Cases: 70 (82.9) Controls: 81 (80.2) Cases: 63.6 (range: 50–75) Controls 64.1 (range: 50–80)	NA	Waist circumference	VAS	Moderate
Sutbeyaz (Turkey, 2007) [109]	Cases were recruited from the musculoskeletal rehabilitation outpatient clinic of Ankara Physical Medicine and Rehabilitation Education and Research Hospital. Controls were nurses, physiotherapist, secretaries, nurse-aids, and maintenance workers.	Cases: 16 (57.1) Controls: 16 (57.1) Cases: 43.96 (10.29) Controls: 43.71 (10.02)	Measuring tape	WHR	WOMAC	High
*Direct fat measurement*						
Sutbeyaz (Turkey, 2007) [109]	Cases were recruited from the musculoskeletal rehabilitation outpatient clinic of Ankara Physical Medicine and Rehabilitation Education and Research Hospital. Controls were nurses, physiotherapist, secretaries, nurse-aids, and maintenance workers.	Cases: 16 (57.1) Controls: 16 (57.1) Cases: 43.96 (10.29) Controls: 43.71 (10.02)	Skin fold callipers	Fat mass	WOMAC	High
Cohort studies						
*Anthropometric fat measurement*						
Pan (Australia, 2020) [101]	Participants recruited from the Tasmanian Older Adult Cohort Study (TASOAC)	Minimal pain: n = 512 (48), 62.9 (7.4) Mild pain: n = 328 (51), 63.0 (7.6) Moderate pain: n = 145 (57), 62.8 (7.2)	NA	WC	WOMAC	Low
Jin (Australia. 2016) [89]	Participants selected from electoral roll	Increase in pain: 175 (54) 62.4 (7.16) No increase in pain: 591 (48) 61.9 (6.97)	Measuring tape	WC, WHR	WOMAC	Low
Batsis (USA, 2014) [91]	Participants recruited form Osteoarthritis Initiative	2182 (60–71 across all groups) 67.5–68.7 across all groups	Measuring tape	WC	WOMAC	Moderate
*Direct fat measurement*						
Pan (Australia 2017) [94]	Tasmania Older Adult Cohort Study	768 (50) 67.1 (7.3)	Dual x-ray absorptiometry	Fat mass, FMI	Presence of pain (yes/no)	Moderate
Jin (Australia. 2016) [89]	Participants selected from electoral roll	Increase in pain: 175 (54) 62.4 (7.16) No increase in pain: 591 (48) 61.9 (6.97)	Dual X-ray absorptiometry	Body fat %	WOMAC	Low
Barber (United States, 2012) [86]	Female basketball players from a single country public school district in Kentucky	248 (100) 12.76 (1.13)	Not specified	Body fat %	Anterior knee pain scale (AKPS) questionnaire	Moderate
**Foot pain**						
Cross-sectional studies						
*Anthropometric fat measurement*						
Butterworth (Australia, 2016) [126]	Individuals selected at random from the electoral roll	796 Foot pain: 68 (IQR: 24–90) No foot pain: 57 (IQR 25–98)	Measuring tape	WHR	MFPDI	Moderate
*Direct fat measurement*						
Butterworth (Australia, 2016) [126]	Individuals selected at random from the electoral roll	796 Foot pain: 68 (IQR: 24–90) No foot pain: 57 (IQR 25–98)	Dual x-ray absorptiometry	Total fat mass FMI	MFPDI	Moderate
Tanamas (Australia, 2012) [119]	From weight loss clinics who range from normal weight to obese	137 (83.2) 47.5 (9.0)	Dual x-ray absorptiometry	Total body, trunk, android & gynoid fat mass, FMI	MFPDI	Moderate
Case control studies						
*Direct fat measurement*						
Walsh (Australia, 2017) [54]	Participants recruited from advertisements placed in newspapers, local general practitioner clinics and online via social media.	88 (100) Cases: 56.6 (10.3) Controls: 56.7 (6.5)	Dual X-ray absorptiometry	Total body fat mass	MFPDI	Moderate
Cohort studies						
*Anthropometric fat measurement*						
Laslett (Australia, 2018) [98]	Participants from Tasmanian Older Adult Cohort study	Foot pain: 227 (55) No foot pain: 333 (49) Foot pain: 63.1 (7.6) No foot pain: 63.0 (7.4)	Not stated	WC	Single question “Do you have pain at any of these sites”	Moderate
*Direct fat measurement*						
Laslett (Australia, 2018) [98]	Participants from Tasmanian Older Adult Cohort study	Foot pain: 227 (55) No foot pain: 333 (49) Foot pain: 63.1 (7.6) No foot pain: 63.0 (7.4)	Dual X-ray absorptiometry	FMI	Single question “Do you have pain at any of these sites”	Moderate
Walsh (Australia, 2018) [99]	Recruited from surgical waiting lists at 2 tertiary hospitals	38 (84) 45.7 (9.4)	Dual X-ray absorptiometry	FMI	Manchester Oxford foot questionnaire	High
Pan (Australia, 2017) [94]	Tasmania Older Adult Cohort Study	768 (50) 67.1 (7.3)	Dual x-ray absorptiometry	Fat mass, FMI	Presence of pain (yes/no)	Moderate
Walsh (Australia, 2016) [90]	The North West Adelaide Health Study (NWAHS)	1462 (53.4) 64.99 (10.58)	Dual x-ray absorptiometry	FMI	Single question “On most days, do you have pain, aching, or stiffness in either of your feet?”	High
Butterworth (Australia, 2013) [85]	Participants from a larger study of obesity and musculoskeletal disease who did not have foot pain at base line	51 (73) 49.2(8.1)	Dual X-ray absorptiometry	FMI & total body fat mass	MFPDI	Moderate

Body fat % = body fat percentage, FMI = Fat mass index, HC = Hip Circumference, LBP = Low Back Pain, MFPDI = Manchester Foot Pain and Disability Index, NHANES = National Health and Nutrition Examination Survey, WC = Waist Circumference, WHR = Waist-Hip Ratio, WHtR = Waist-height Ratio, WOMAC = Western Ontario and McMaster Universities Arthritis Index. VAS = visual analogue scale

### Study populations

A total of 39 studies recruited both male and female participants [6,85,87–94,96–101,107,109,110,112–115,117–119,121,122,125,127–131,134–138], while eleven studies included female participants only [7,86,102,104–106,108,116,120,132,133], five studies included male participants only [95,103,111,123,124] and one study did not specify the gender of their participants [126] (Table 1). The mean age of the participants in 41 studies was above 40 years [6,7,85,89–94,97–99,101–111,113,116,118–121,123,125,126,128–133,135–137], while six studies had a mean participant age between 20–40 years [95,115,124,127,134,138], and three studies had a mean age below 18 years [86,117,123]. Bihari et al. [114] included participants from 10 to 70 years of age, Brooks et al. [122] included participants from 18–76 years, Shiri et al. [96] included participants over the age of 30, Heuch et al. [88] included participants from 30–69 years and Muthuri et al. [100] followed participants over 32 years, collecting data at the age of 36, 43, 53, 60–64 and 68–69 years. One study did not specify the age of their participants [112].

Participant data were obtained from 12 existing databases or studies; including the Osteoarthritis Initiative [91], Australian Diabetes, Obesity and Lifestyle Study [92], National Health And Nutrition Examination Study [112], Western Australian Pregnancy Cohort [117], Young Finns Study [87,127], Morgan project [113], Nord-Trøndelag Health Study [88], Hong Kong Department of Community and Family Medicine study [104], North West Adelaide Health Study [90], Tasmania Older Cohort Study [94,98,129], PAINEL study [130], CoHRE study [131], Tasmanian Older Adult Cohort Study (TASOAC) [101], a clinical trial of vitamin D in overweight/obese individuals [134], Yakumo study [132] and a British cohort study based on the MRC National Survey of Health and Development [100]. Participants were also recruited from local GP or health care clinics in eight studies [6,102,106,116,118–120,138] and from hospitals, and outpatient and rehabilitation clinics in seven studies [107,109–111,115,136,137] and registries in four studies [93,105,123,124]. Three studies recruited from electoral role [7,89,126], three studies recruited from media advertising and leaflet drops [85,97,122], and three studies from surveys [96,125,135], two studies recruited from government offices and schools [108,133], and single studies recruited from a physical therapy department [128], companies in the metropolis area [95], the national capital region [114], an annual medical examination [121], surgical waiting list [99], a municipal transport company [103] and a country public school [86].

### Assessment of adiposity

Adiposity was assessed using various methods; 16 studies used dual energy X-ray absorptiometry [6,7,85,89,90,94,97–100,106,107,119,125,126,129], 11 studies used bioelectric impedance analysis [92,93,105,114,118,120–122,128,131,137], 20 studies used a tape measure to determine waist and hip circumference [87,89,91–93,102,104,105,109,112,113,116,117,122,126,127], two studies used mathematical calculations [103,116,132,135,137,138], and five studies used skin fold callipers [95,108,109,111,115] (Table 1). Twelve studies did not specify how adiposity was measured [86,88,96,100,101,110,123,124,130,133,134,136].

Different adiposity measures were reported across the studies, with 18 studies measuring body fat percentage [86,89,92–95,105,107,108,114–116,118,120,128,129,134,137], 20 studies measuring fat mass [6,7,85,90,92,97–100,107,109,119–122,125,126,131,134,137], 29 studies measuring waist and/or hip circumference [87–89,91–93,96,98,100–105,110,112,113,116,117,123,124,127,130,132–136,138], 13 studies measuring waist-hip ratio [7,87,93,105,109,113,116,118,122,127,132,133,138], two studies measuring waist height ratio [46,133] and one study measuring percentage of body fat volume [111].

### Assessment of pain

A range of measures were used to assess pain (Table 1). While the Western Ontario and McMaster Universities Arthritis Index (WOMAC) and visual analogue scale (VAS) were the most commonly used validated tools, a large number of studies used structured interviews or self-administered questionnaires. Low back pain was examined using the visual analogue scale [116,121,122,132,137,138], Chronic Pain Grade scale [6,7,92,97], NHANES general wellbeing index [112], Nordic Musculoskeletal Questionnaire [133], questions regarding the history of low back pain (e.g. Have you ever had back pain? (“yes” or “no”)) [88,93,95,96,100,104,105,107,108,111,113,115,117,123,130,131,134–136] and structured interviews [103,114,127]. Hip pain was assessed by asking about the presence of pain (yes/no) [94] and any history of hip pain [102]. Knee pain was assessed using WOMAC Index [89,91,101,109,128], questions regarding the presence of pain (yes or no) [94,129], anterior knee pain scale [86] and visual analogue scale [110,120,132] and self-administered questionnaires [124,125]. Foot pain was measured using the Manchester Foot Pain and Disability Index [85,106,119,126], Manchester-Oxford foot questionnaire [99] and asking about the presence of pain (yes/no) [94,98], or the history of foot pain (Over the past month, have you had pain, aching, or stiffness in either of your feet on most days?) [90].

The follow-up periods between baseline and the assessment of pain varied between the cohort studies. Of the 8 cohort studies of back pain [87,88,92,93,95–97,100], the follow-up time ranged from 2 to 20 years, with half of the studies investigating time periods less than 10 years and half of the studies examining time periods over 10 years. The single cohort study of hip pain followed up participants over 5 years [94], while the 5 cohort studies of knee pain ranged from 2 to 10.7 years [86,89,91,94,101], with 3 studies examining time periods of 5 or 6 years. Moreover, the 5 studies examining foot pain had follow-up periods ranging from 4 to 20 years [85,90,94,98, 99], with 4 of the 5 studies focusing on a 3–5 year follow-up.

### Risk of bias assessment

Of the 56 studies included in the review, 24 had a high risk of bias [86–88,90,95,99,102–105,107–109,111–116,118,124,127,130,137], 24 had a moderate risk of bias [6,7,85,91–94,96–98,100,106,110,117,119–121,123,128,131,134–136,138], and eight had a low risk of bias [89,101,122,125,126,129,132,133] (Table 1). Of the 17 cohort studies, the risk of bias was rated as high for six studies [86–88,95,99,106,111,137] and low to moderate for eleven studies [85,89,91–94,96–98,100,101]. For these cohort studies, the criteria ‘assessment of exposure’ and ‘assessment of outcome’ more frequently scored a high risk than the other Cochrane criteria. Eight of the ten case-control studies were assessed as having a high risk of bias [102–105,107–109,111], and two a moderate risk of bias [106,110]. The criteria ‘assessment of exposure’ and ‘assessment of outcome’ were most frequently associated with high risk of bias when assessing the case-control studies. Of the 29 cross sectional studies, ten had a high risk of bias [112–116,118,124,127,130,137], 13 had a moderate risk of bias [6,7,117,119–121,123,128,131,134–136,138], and six had a low risk of bias [122,125,126,129,132,133]. The criteria associated with the ‘assessment of the outcome’ were most frequently associated with a high risk of bias for cross-sectional studies.

### Relationship between adiposity and low back pain

#### Anthropometric fat measures

*Waist circumference*. Twenty one studies examined the association between waist circumference and low back pain (Table 2). Of these studies, 13 were cross sectional studies [112,113,116,117,123,127,130,132–136,138], two were case control studies [104,105] and six were cohort studies [87,88,92,93,96,100]. Eight of the 13 cross-sectional studies found significant associations between waist circumference and low back pain [112,113,117,127,132,134–136], with two studies reporting an association in females only [113,127], two studies finding a relationship in males only [117,136] and the remaining 4 studies finding an association in both males and females [112,132,134,135]. Five studies did not find an association between waist circumference and radiating and non-specific low back pain [123], presence of low back pain [130,133] or low back pain intensity [116,138]. Of the two case-control studies, one study found greater waist circumference was associated with less low back pain (lasting 14 days or greater) in middle age women [104], while the other study found no association between waist circumference and chronic low back pain [105].

**Table 2:** Results of the studies investigating the relationship between adiposity and low back pain.

First author (year)	Definition of pain	Variables adjusted for	Main findings (OR/RR/β coefficient (95% CI))	Conclusions
**Low back pain**				
Cross-sectional studies				
*Anthropometric fat measurement*				
Yoshimoto (2019) [136]	Responded yes to the question, “Do you have LBP under treatment including follow-up?”	Adjustment for age, smoking habits, alcohol intake, and physical activity.	**Men:** **Abdominal obesity:** LBP vs no LBP: 335 (52.3) vs 13,709 (45.6) **No abdominal obesity:** LBP vs no LBP: 306 (47.7) vs 56,345 (54.4) Pearson’s chi square: p < 0.001. OR: 1.34 (1.02, 1.76) **Women:** **Abdominal obesity:** LBP vs no LBP: 52 (21.1) vs 1749 (12.3) **No abdominal obesity:** LBP vs no LBP: 194 (78.9) vs 12,502 (87.7) Pearson’s chi square: p < 0.001. OR: 1.70 (0.94, 3.08)	The proportion of abdominal obesity was significantly higher in participants with LBP than in those without LBP for each sex. The presence of abdominal obesity was significantly associated with LBP among men, but not among women.
Hussien (Egypt, 2019) [138]	Continuous or recurrent localised LBP ≥ 3 months. Pain intensity rating on the VAS of ≥ 1.	No adjustments made.	WC: T_b_ = -0.02, p = 0.7 HC: T_b_ = 0.04, p = 0.6 WHR: T_b_ = -0.04, p = 0.5	There were no associations between the anthropometric measures and pain intensity.
Kulandaivelan (2018) [135]	Pain lasting > 1 day in the past 12 months	NA	**Presence of pain:** OR: 1.39 (1.08, 1.81)	Abdominal obesity increases the risk of low back pain.
Brady (2018) [33]	Responded yes to LBP in the past month.	Age, sex	**Presence of pain** WC 109.6 ± 16.8 vs 101.0 ± 9.3 cm OR: 1.1 (1.0, 1.1)	Participants who had back pain in the past month had a higher waist circumference compared to those without back pain.
Machado (2018) [130]	Disabling pain in the last 1 year	Gender, BMI, WC, self-rate health, multi-morbidity, chronic musculoskeletal pain other than LBP, frequent LBP, physical activity, low gait speed, fatigue, sitting, sleep, depression symptoms, depression diagnosis, fear beliefs	**Presence of disabling LBP** **WC (male ≥102 cm, female ≥88)** OR 0.47 (0.11–2.14)	WC, dichotomised into high and low, was not associated with LBP.
Ogwumike (Nigeria, 2016) [133]	Presence of back pain in the past year.	Age	**Presence of pain** WC: 1.51 (0.94, 2.40) WHtR: 1.70 (1.07, 2.75) WHR: 1.04 (0.66, 1.67)	Waist height ratio (WHtR) was found to be associated with LBP in post-menopausal women.
Chou (2016) [7]	Cohort split into two groups: 1. No pain/disability or low intensity pain (<50) and low disability (<3) 2. High intensity pain (≥50) or high disability (≥3)	Age, emotional disorder, education and mobility	**High-intensity pain and/or disability vs low-intensity pain and/or disability** (Estimated marginal means) WHR (SD): 0.96 (0.006) vs 0.97 (0.006), p = 0.04	WHR was higher in those with either high intensity pain or high disability compared to those with no or low intensity pain or no or low disability.
Frilander (2015) [123]	Yes response to chronic LBP and radiating LBP (0 = no, 1 = below knee, 2 = above knee)	Age, smoking, education	**Chronic LBP** (WC, <94 cm ref) 94–101.9cm: OR 1.04 (0.63–1.73) ≥102cm: OR 1.24 (0.75–2.03) **Radiating LBP** (WC <94 cm ref) 94–101.9cm: OR 1.03 (0.69–1.53) ≥102cm: OR 1.31 (0.88–1.96)	WC was not associated with incident, chronic LBP. WC was not associated with incident, radiating LBP.
			**Chronic LBP** (Waist-to-height ratio, ≤0.5 ref) >0.5: OR 1.33 (0.75–1.72) **Radiating LBP** (Waist-to-height ratio, ≤0.5 ref) >0.5: OR 1.44 (1.02–2.04)	
Muramoto (2014) [132]	Pain intensity rating on the VAS of ≥ 1.	Age	WC: r = 0.2, p<0.005 HC: r = 0.2, p<0.01 WHR: r = 0.2, p<0.01 Multivariate analyses: WC: significant association reported. Data not provided. p<0.05.	Central obesity was associated with LBP intensity.
Briggs (2013) [112]	Positive response to “during the past 3 months, did you have LBP?”	NA	**LBP vs no LBP** Men (WC, <102 cm ref) vs women (WC, <88 cm ref) Chi-squared; p<0.001	A larger WC increased the odds of reporting LBP.
Ojoawo (2011) [116]	Pain rating between 1 and 10.	NA	**Pain intensity** HC: r = 0.41, p<0.05 WC: r = 0.24, p>0.05 WHR: r = 0.18, p>0.05	Increased HC, but not WC or WHR had a significant relationship with the intensity of pain experienced in women with low back pain.
Perry (2009) [117]	Positive response to back pain ever, back pain in past month or chronic back pain lasting >3 months.	Physical characteristics	**Prevalent LBP Male (IQR ref)** Univariate Low WC: OR 0.45 (0.23–0.86) High WC: OR 1.12 (0.67–1.86) Multivariate WC: OR 2.20 (1.11, 4.36)	An increased likelihood of low back pain was associated with greater central adiposity in adolescent males but not females.
			**Prevalent LBP Female (IQR ref)** Univariate: Low WC: OR 1.14 (0.68–1.93) High WC: OR 1.06 (0.62–1.81) WC: OR Data not provided.	
Shiri (2008) [127]	Dichotomous variable of LBP. Those who recovered in one month, had recurrent or continuous back pain compared with those who recovered in one week or had no back pain.	Age, educational status, occupational status and smoking	**Pain intensity** **Male (WC, <94.0cm ref)** 94.0–101.9cm: OR 1.1 (0.7–1.6) ≥102.0cm: OR 0.7 (0.4–1.1) **Female (WC, <80.0cm ref)** 80.0–87.9cm: OR 1.3 (0.9–1.8) ≥88.0cm: OR 1.8 (1.3–2.4)	WC, HC and WHR were significantly associated with LBP in females, but not in males.
			**Pain intensity** **Male (HC, lowest tertile ref)** Middle tertile: OR = 1.3 (0.9–1.9) Highest tertile: OR = 1.0 (0.6–1.4) **Female (HC, lowest tertile ref)** Middle third: OR = 1.0 (0.7–1.4) Highest third: OR = 1.6 (1.1–2.1)	
			**Pain intensity** **Male (WHR, <0.9 ref)** 0.9–1.0: OR 0.9 (0.5–1.8) >1.0: OR 0.9 (0.5–1.8) **Female (WHR, <0.8 ref)** 0.8–0.9: OR 1.2 (0.8–1.5) >0.9: OR 2.3 (1.3–3.9)	
Toda (2000) [118]	Responded ‘yes’ to duration of current episode of LBP > 3 months or recurrent LBP compared to responded ‘no’ to LBP or low back problems in past 10 years.	NA	**Presence of pain** **Female (WHR)** Control vs negative straight leg raise 86.5 (5.3) vs 90.8 (6.4), p<0.001 Control vs positive straight leg raise 86.5 (5.3) vs 87.3 (6.3), p>0.05	Central adiposity may be a risk factor for chronic low back pain with a negative straight leg raise test result in women, but not in men. Positive straight leg raise was not associated with central adiposity.
			**Presence of pain** **Male (WHR)** Control vs negative straight leg raise 90.2 (4.4) vs 90.5 (4.8), p>0.05 Control vs positive straight leg raise 91.9 (4.0) vs 90.5 (4.8), p>0.05	
Han (1997) [113]	Responded yes to LBP in past 12 months. Chronic LBP defined as responded yes to a total of twelve weeks or more.	Age, smoking, education	***WC****tertiles 86*.*9cm and 95*.*9cm for males*, *75*.*0cm and 84*.*0cm for females*, *lowest tertile ref*. **Male (chronic LBP)** Middle tertile: OR 0.94 (0.78–1.14) Highest tertile: OR 1.13 (0.94–1.37) **Male (LBP past 12 months)** Middle tertile: OR 0.89 (0.78–1.02) Highest tertile: OR 0.97 (0.85–1.12) **Female (chronic LBP)** Middle tertile: OR 1.26 (1.08–1.48) Highest tertile: OR 1.49 (1.27–1.75) **Female (LBP past 12 months)** Middle tertile: OR 1.12 (1.00–1.27) Highest tertile: OR 1.21 (1.06–1.37)	Women with a large waist (increased central adiposity) have a significantly increased likelihood of low back pain. There was no association for men.
			***WHR****tertiles 0*.*872 and 0*.*936 for males*, *0*.*756 and 0*.*815 for females*, *lowest tertile* **Male (chronic LBP)** Middle tertile: OR 0.93 (0.77–1.13) Highest tertile: OR 0.98 (0.80–1.19) **Male (LBP past 12 months)** Middle tertile: OR 0.97 (0.85, 1.11) Highest tertile: OR 1.00 (0.79–1.06) **Female (chronic LBP)** Middle tertile: OR 1.27 (1.09–1.50) Highest tertile: OR 1.35 (1.15–1.58) **Female (LBP past 12 months)** Middle tertile: 1.02 (0.91–1.15) Highest tertile: 1.14 (1.01–1.30)	
*Direct fat measurement*				
Endo (2019) [131]	Responded yes to “Do you have any low back pain at present?”	NA	**Female (No LBP vs LBP)** Fat mass, kg (SD): 15.2 (6.4) vs 15.4 (6.2), p = 0.55 **Male (No LBP vs LBP)** Fat mass, kg (SD): 11.7 (5.0) vs 11.9 (5.1), p = 0.51	There were no significant differences in fat mass between participants with LBP and without LBP, in either female or male groups.
Brady (2018) [33]	Responded yes to LBP in the past month.	Age, sex	**Presence of pain** Fat mass 39.9 ± 12.3 vs. 33.9 ± 9.8%, p = 0.04 OR: 1.1 (1.0, 1.1)	Participants who reported having back pain in the past month had higher fat-mass compared to those without back pain.
Nava-Bringas (2018) [137]	Chronic low back pain (>3 months) and radiographic evidence of lumbar osteoarthritis (including facet joint osteoarthritis and disc degeneration). Pain score of ≥1 over the past 7 days.	No adjustments made.	**Pain intensity** Fat mass rho: -0.239 p = 0.2 % body fat rho: 0.09 p = 0.7	There was no correlation between fat mass or percentage body fat and back pain.
Brooks (2016) [122]	VAS score, minimum of 2.0 and maximum of 10.0.	NA	**Pain intensity** Abdominal to lumbar fat mass ratio r = 0.32, p = 0.007	Abdominal adiposity was associated with chronic LBP.
Chou (2016) [7]	Cohort split into two groups: 1. No pain/disability or low intensity pain (<50) and low disability (<3) 2. High intensity pain (≥50) or high disability (≥3)	Age, emotional disorder, education and mobility	**No or Low-Intensity Pain/Disability vs High-Intensity Pain and/or Disability** (Estimated marginal means) Fat mass, kg (SD): 23.2 (0.3) vs 24.5 (0.7), p = 0.10 FMI, kg/m^2^ (SD): 7.6 (0.1) vs 8.0 (0.2), p = 0.08	There were no significant differences in fat mass or FMI between those with no or low intensity pain/disability compared with those with high intensity pain/disability.
Iizuka (2015) [121]	Incident pain defined as responded yes to “Do you have low back pain at present?” Chronic pain defined as responded yes to “Have you had chronic low back pain persisting for three months or more?”	Age, gender	**Presence of pain** (total body fat mass) OR 1.02 (0.94, 1.02) **Chronic pain** (total body fat mass) OR 0.98 (0.93, 1.03) **Presence of pain plus intensity** (total body fat mass) β -0.05 (-0.07, 0.03)	Total body fat mass did not have a significant relationship with incidence, chronicity or intensity of present LBP.
Bihari (2011) [14]	Not specified	No adjustments	Backache vs no musculoskeletal disorders (total body fat mass) OR 1.2 (0.9–1.7)	Higher body fat percentage was not associated with back pain.
Ojoawo (2011) [116]	Pain rating between 1 and 10.	NA	**Pain intensity** (body fat %) r = 0.67, p<0.01	Increased body fat percentage has a significant relationship with the intensity of pain experienced in women with low back pain.
Urquhart (2011) [6]	Pain intensity measured on the Chronic Pain Grade Questionnaire, 0–100. Low pain intensity <50 High pain intensity ≥50	Age, sex, height, physical activity, fat or lean tissue	**Pain intensity** (total fat mass) OR 1.19 (1.04–1.38) **Pain intensity** (upper limb fat mass) OR 1.18 (0.93–1.50) **Pain intensity** (lower limb fat mass) OR 1.51 (1.04–2.20)	Greater total and lower limb fat mass were associated with higher levels of low back pain intensity.
Hodselmans (2010) [115]	Responded yes to LBP for >3 months.	NA	**Predicted normative data vs LBP participants** (fat mass %) Mean (SD): 26.4 (6.1) vs 30.4 (8.2) p<0.001	Patients with chronic LBP have an increased body fat percentage.
Toda (2000) [118]	Responded ‘yes’ to duration of current episode of LBP > 3 months or recurrent LBP compared to responded ‘no’ to LBP or low back problems in past 10 years.	NA	**Presence of pain** Female (body fat %) Control vs negative straight leg raise 27.9 (6.7) vs 30.5 (6.5), p = 0.03 Control vs positive straight leg raise 28.6 (7.0) vs 27.9 (6.7), p>0.05	Body fat mass percentage may be a risk factor for chronic LBP without a positive straight leg raise test result in women, but in not men. Positive straight leg raise was not associated with central adiposity.
			**Presence of pain** Male (body fat %) Control vs negative straight leg raise 22.6 (5.7) vs 22.3 (6.1), p>0.05 Control vs positive straight leg raise 24.9 (4.4) vs 22.3 (6.1), p>0.05	
Case control studies				
*Anthropometric fat measurement*				
Dario (2016) [105]	Responded yes to “Have you ever suffered from chronic LBP?” with chronic defined as lasting at least 6 months.	Smoking, leisure physical activity	**Presence of pain** Chronic LBP (WC) OR 1.06 (0.93–1.22) Chronic LBP (WHR) OR 1.02 (0.89–1.17)	WC and WHR are not associated with chronic LBP.
Yip (2001) [104]	Not specified	Source of recruitment (random subjects from population-based study vs family clinic subjects), menopausal status	**LBP ≥ 1 day** WC: OR 0.67 (0.41–1.09) HC: OR 0.80 (0.49–1.31) WHR: OR 0.72 (0.47–1.11) **LBP ≥ 14 days** WC: OR 0.52 (0.29–0.92) HC: OR 1.10 (0.62–0.70) WHR: OR 0.43 (0.26–0.70)	High WHR ratios was inversely associated with the risk of severe LBP in middle-aged women.
Hultman (1993) [111]	3 groups: Group 1: never had LBP or slight LBP impairment Group 2: had several or at least one episode of LBP, no LBP for 2 months pre-study Group 3: ≥3 years of chronic LBP, > 3 months of sick leave in the previous year	NA	**Presence of pain** Fat volume % (skin folds) Group 1: 30 (6) Group 2: 28 (6) Group 3: 28 (6) Data not provided.	There were no differences in fat volume between those with no, intermittent and chronic LBP.
**Direct fat measures**				
Sakai (2017) [107]	Persistent moderate to severe LBP for minimum previous 3 months	NA	**Male (LBP vs no LBP)** Upper limb fat mass (g) 1781.18 ± 728.75 vs 1655.43 ± 656.38, p = 0.24 Lower limb fat mass (g) 4509.52 ± 1530.68 vs 4054.76 ± 1391.11, p = 0.05 Body fat % 35.77 ± 6.71 vs 27.69 ± 7.57, p<0.001	Body fat percentage was significantly higher in participants with LBP in both male and females. Additionally, lower limb fat mass was significantly greater in males with LBP.
			**Female (LBP vs no LBP)** Upper limb fat mass 1978.41 ± 553.97 vs 2053.18 ± 998.24, p = 0.59 Lower limb fat mass 4902.61 ± 1338.75 vs 4861.08 ± 1826.70, p = 0.87 Body fat % 41.05 ± 4.09 vs 34.25 ± 8,84, p<0.001	
Dario (2016) [105]	Responded yes to “Have you ever suffered from chronic LBP?” with chronic defined as lasting at least 6 months.	Smoking, leisure physical activity	**Presence of pain** Chronic LBP (body fat %) OR 1.15 (1.01–1.32)	Body fat percentage was associated with LBP prevalence in women.
Spyropoulos (2008) [108]	Complained about LBP symptoms for a minimum of 15 months.	NA	**Healthy women vs women with chronic LBP** (body fat %) 31.3 (5.2) vs 34.7 (5.1), p = 0.035	Body fat percentage was significantly higher in women working in offices with chronic LBP compared to healthy controls.
Celan (2005) [103]	Responded yes to previous LBP and also responded yes to having 3 times or more previous episodes.	NA	**No low back problems vs recurrent low back problems** (body fat %) 25.54 vs 26.39, p = 0.43	There were no significant differences in body fat percentage between those with and without low back pain.
Cohort				
**Anthropometric fat measures**				
Muthuri (2020) [100]	All ages (except 68 yo): Responded yes to the question about whether they had sciatica, lumbago or recurring/severe backache all or most of the time (ever at ages 36 and 43 and in the previous 12 months at ages 53 and 60–64). Age 68: Responded yes to the question about whether they had experienced any ache or pain in the previous month which had lasted for 1 day or longer.	Age, BMI, sex, education, occupational class and time-varying covariates (height, cigarette smoking status, physical activity and symptoms of anxiety and depression).	**Follow-up** 36 to 43 yo: 7 years 43 to 53 yo: 10 years 53 to 60–64 yo: 7–10 years 60–64 to 68 yo: 4–8 years **Presence of pain** 36 years: 1.08 (0.97, 1.21) 43 years: 1.14 (1.02, 1.26) 53 years: 1.23 (1.07, 1.40) 60–64 years: 1.06 (0.92, 1.21)	Higher WC was associated with increased odds of back pain between the ages 36 and 68.
Shiri (2019) [96]	Assessed by the number of days of LBP in the last 12 months	Age, gender, BMI, physical activity, walking or cycling to work, depression, strenuous physical work, using vibrating tools, keeling or squatting, standing or leaning forward, LBP past 30 days	**11 year follow-up** **Presence of pain** LBP > 7 days (WC, normal ref) Increased: OR 1.07 (0.88–1.31) Obese: OR 1.40 (1.16–1.68) LBP > 30 days (WC, normal ref) Increased: OR 0.98 (0.77–1.26) Obese: OR 1.41 (1.13–1.76)	Individuals with an obese WC were at a higher risk of larger number of days of LBP than those with a normal WC.
Dario (2017) [93]	Responded yes to “Have you ever suffered from chronic LBP?”	Age, gender	**2 to 4 year follow-up:** **Incident chronic LBP** Total sample (WC, no pain ref) OR 0.98 (0.74–1.30) Within MZ and DZ twins (WC, no pain ref) OR 0.48 (0.16–1.50) Total sample (WHR, no pain ref) OR 1.05 (0.81–1.36) Within MZ and DZ twins (WHR, no pain ref) OR 0.47 (0.18–1.21)	Risk of chronic back pain was no higher for individuals with an increased WC or WHR.
Hussain (2017) [92]	Pain categorised into 3 groups from the Chronic Pain Grade Questionnaire; no pain (0), low pain (<50), high pain (≥50)	Age, education, smoking status, socio-economic indexes for areas, mental component score of SF-36	**12 year follow-up:** Male (WC, no pain ref) **Low intensity LBP** OR 1.11 (0.98–1.27) **High intensity LBP** OR 1.25 (1.07–1.46)	A larger WC was significantly associated with high intensity LBP compared to no pain in both male and females. A larger WC was also significantly associated with low intensity LBP in females, but not in males. When WC was split into quartiles, each quartile had significantly higher pain levels compared to the lowest WC quartile in both males and females, except for quartile 2 in females.
			Female (WC, no pain ref) **Low intensity LBP** OR 1.13 (1.03–1.24) **High intensity LBP** OR 1.36 (1.22–1.52)	
			**12 year follow-up:** **Pain intensity** Male (WC, quartile 1 ref) Quartile 2: OR 1.43 (1.10–1.84) Quartile 3: OR 1.78 (1.36–2.34) Quartile 4: OR 1.50 (1.12–2.00)	
			**Pain intensity** Female (WC, quartile 1 ref) Quartile 2: OR 1.20 (0.96–1.50) Quartile 3: OR 1.42 (1.13–1.78) Quartile 4: OR 2.09 (1.65–2.65)	
Heuch (2015) [88]	Responded yes to “During the last year, have you had pain and/or stiffness in your muscles and limbs that has lasted for at least 3 consecutive months?” and responded lower back to “Where did you have pain and/or stiffness?”	Age, education, work status physical activity, smoking, HDL-cholesterol, triglycerides, blood pressure, body weight, BMI, WC, HC	**11 year follow-up:** Female **Incident LBP** WC: RR 1.08 (1.03–1.13) WHR: RR 1.03 (0.99–1.08) HC: 1.07 (1.02, 1.12) **Recurrent or persistent LBP** WC: RR 1.07 (1.04–1.10) WHR: RR 1.02 (0.99–1.05) HC: 1.07 (1.04, 1.10)	WC was associated with recurrence/persistence and incidence of pain in women but not in men. WHR was not associated with LBP in women or men.
			Male **Incidence LBP** WC: RR 1.06 (1.00–1.13) WHR: RR 1.04 (0.98–1.10) HC: 1.06 (1.00, 1.12) **Recurrence or persistent LBP** WC: RR 1.02 (0.97–1.07) WHR: RR 1.01 (0.97–1.06) HC: 1.02 (0.97, 1.06)	
Shiri (2013) [87]	Responded yes to “Have you had low back trouble (pain, ache, or unpleasant sensations) during the preceding 12 months?” and responded greater than 7 days to “What is the total length of time you have had low back trouble during the preceding 12 months?”	Age, gender, educational status, occupational status, smoking	**6 year follow-up:** **Presence of non-specific LBP > 7 days** (WC baseline, normal ref) *Male* Increased: OR 1.1 (0.6–2.0) Obese: OR 0.9 (0.5–1.8) 1cm increase: OR 1.00 (0.98–1.03) *Female* Increased: OR 1.7 (0.9–2.8) Obese: OR 1.1 (0.6–2.0) 1cm increase: OR 1.01 (0.99–1.03)	Baseline WC and average WC over 7 years were not associated with non-specific LBP in males or females, with the exception of an obese WC in females.
			**Presence of non-specific LBP** (WC 7 year average, normal ref) *Male* Increased: OR 0.8 (0.5–1.5) Obese: OR 0.8 (0.4–1.6) 1cm increase: OR 1.00 (0.98–1.02) *Female* Increased: OR 1.5 (0.9–2.6) Obese: OR 1.7 (1.0–3.0) 1cm increase: OR 1.01 (0.99–1.04)	
*Direct fat measurement*				
Muthuri (2020) [100]	60–64 years: Responded yes to the question about whether they had sciatica, lumbago or recurring/severe backache all or most of the time in the previous 12 months. 68 years: Responded yes to the about whether they had experienced any ache or pain in the previous month which had lasted for 1 day or longer.	Sex, lean mass index, fat mass index, education at age 26, occupational class at age 53 and the following covariates (assessed at age 60–64): height, cigarette smoking status, physical activity and symptoms of anxiety and depression	**Follow-up:** 60–64 to 68 yo: 4–8 years **Presence of pain:** FMI OR: 1.24 (1.04, 1.45)	Higher fat mass index was associated with higher odds of back pain at age 68.
Brady (2019) [97]	High pain intensity >50 out of 100	Age, gender, strenuous physical activity, mental health component score, total lean tissue mass	**3 year follow-up:** **Pain intensity** Fat mass: OR 1.05 (1.01–1.09)	Individuals with greater fat mass had a greater risk of high intensity LBP.
Dario (2017) [93]	Responded yes to “Have you ever suffered from chronic LBP?”	Age, gender	**2–4 year follow-up:** **Incident chronic LBP** Total sample (percent fat, no pain ref) OR 0.87 (0.66–1.14) Within MZ and DZ twins (percent fat, no pain ref) OR 1.00 (0.35–2.85)	Percentage fat mass was not predictive of LBP in adult twins.
Hashimoto (2017) [95]	Did not have LBP in the past or the present at baseline.	Maximal oxygen uptake, age, drinking, smoking	**20 year follow-up:** **Incident LBP** Persistent LBP per 10,000 men years (body fat percentage quartiles) Q1: reference Q2: OR 0.86 (0.43–1.71) Q3: OR 1.46 (0.79–2.72) Q4: OR 2.12 (1.13–3.98)	Individuals within the highest quartile of body fat mass were more likely to develop LBP compared to those in the lowest quartiles.
Hussain (2017) [92]	Pain categorised into 3 groups from the Chronic Pain Grade Questionnaire; no pain (0), low pain (<50), high pain (≥50)	Age, education, smoking status, socio-economic indexes for areas, mental component score of SF-36	**12 year follow-up:** **Pain intensity** Male (no pain ref) Low intensity LBP (percent fat) OR 1.28 (1.09–1.51) High intensity LBP (percent fat) OR 1.45 (1.19–1.77) Low intensity LBP (fat mass) OR 1.11 (0.97–1.27) High intensity LBP (fat mass) OR 1.23 (1.05–1.44)	Both males and females with a higher percentage fat mass and total fat mass were at higher risk of high intensity LBP compared to individuals with no pain. Individuals with a higher percentage fat mass were at higher risk of low intensity LBP compared to those with no pain. Females, but not males, with larger total fat mass were at higher risk of low intensity LBP compared to no pain.
			**12 year follow-up:** **Pain intensity** Female (no pain ref) Low intensity LBP (percent fat) OR 1.41 (1.25-.1.59) High intensity LBP (percent fat) OR 1.39 (1.22–1.57) Low intensity LBP (fat mass) OR 1.28 (1.16–1.41) High intensity LBP (fat mass) OR 1.27 (1.15–1.40)	

DZ = dizygotic, HC = hip circumference, IQR = inter-quartile range, LBP = low back pain, MZ = monozygotic, NA = not available, OR = odds ratio, RR = relative risk, SD = standard deviation WC = waist circumference, WHR = waist-hip ratio.

Of the six cohort studies, five studies found significant associations [87,88,92,96,100]. Three studies found a significant relationship between waist circumference and the presence of low back pain [87,88,100], with two studies reporting obese waist circumference to be associated with a larger number of days of low back pain [114,117] and one study finding waist circumference to be associated with high intensity low back pain [92]. The other two studies examined the relationship between waist circumference and incident low back pain and reported conflicting results [93], with one of the studies also examining recurrent and persistent low back pain and finding an association with waist circumference [59].

*Hip circumference*. Six studies examined the association between hip circumference and low back pain. Of the six studies, three cross-sectional studies found significant associations between hip circumference and low back pain intensity [116,132], but one study reported it in females only [127]. However, one cross-sectional study reported no significant association between hip circumference and low back pain intensity, a case-control study found no significant association between hip circumference and low back pain defined as pain for one or more days or 14 or more days [104] and one cohort study found no association between hip circumference and incident or recurrent/persistent low back pain [88].

*Waist-hip ratio and waist-to-height ratio*. Twelve studies, including eight cross sectional, two case-control and two cohort studies, examined the association between waist-hip ratio and low back pain. Of the eight cross-sectional studies [7,86,113,118,127,132,133,138], {], five found an association between waist-hip ratio and low back pain [7,113,118,127,132], while three did not find a relationship [86,133,138]. One case control study found waist-hip ratio was significantly associated with low back pain for 14 or more days [104], while another case control study found no association [105]. Both cohort studies found no association between waist-hip ratio and incident low back pain, with one study investigating this relationship in twins [93], and the other study examining females and males separately [88].

With respect to waist-to-height ratio, two studies examined the relationship between this adiposity measure and low back pain, finding an association with the presence of pain in post-menopausal women [133], and with radiating low back pain, but not chronic low back pain [123].

#### Direct fat measures

*Body fat mass*. Twelve studies, including eight cross sectional, one case-control and three cohort studies, examined the association between body fat mass and low back pain (Table 2). Of the eight cross sectional studies, one study found an association between abdominal to lumbar fat mass ratio and low back pain [122], and three studies found an association between total body fat mass and pain intensity [6,7] and the presence of low back pain [134]. The remaining four studies found no association between total body fat mass and chronic low back pain [114,121,131,137]. The case control study found an association between fat mass and presence of pain in males, but not females [107]. While one cohort study found associations between fat mass and high intensity pain in females and males, and fat mass and low intensity pain in females only [92], the remaining two cohort studies found greater fat mass was associated with a higher risk of the presence of pain [100] and high pain intensity [97].

*Body fat percentage*. Twelve studies, including five cross sectional, four case-control and three cohort studies, examined the association between body fat percentage and low back pain. Three cross sectional studies found associations between body fat percentage and low back pain [115,116,118], while two found no association [134,137]. Three case control studies found significant associations between body fat percentage and chronic low back pain [105,108] and presence of pain [107], while the remaining case control study found no association between body fat percentage and recurrent low back pain [103]. One cohort study found percentage fat mass to be significantly associated with high intensity pain in both females and males [92]. The second cohort study found those in the highest quartile of body fat mass were significantly more likely to develop low back pain than those in the lowest quartile [95] and the third study found no associations between percentage fat mass and incident low back pain in twins [93].

#### Summary of the evidence

Overall there was evidence of an association between adiposity and low back pain from 26 of the 36 identified studies (Table 3). Specifically, there was evidence from 5 of 6 cohort studies and 12 of 15 cross-sectional studies to indicate that there is a positive relationship between central adiposity and low back pain. There was also evidence provided by six of six cohort studies for a longitudinal relationship between adiposity and presence of low back pain, but conflicting evidence for a relationship between adiposity and incident low back pain (two of three studies) and limited evidence for a relationship with increasing low back pain (one of one study) (Table 4).

**Table 3:** Summary of the evidence examining the relationship between any and central adiposity and back and lower limb pain.

		No. of studies using direct and anthropometric measures: Any adiposity			No of studies using anthropometric measures: Central adiposity^		
	Conducted	Association	No association		Association	No association	
*Low back pain*				Evidence			Evidence
Cohort	8	7	1		5	1	
Case Control^^	6	4	2		-1*	2	
Cross-sectional	22	15	7		12**	3	
*Hip pain*				Limited evidence			Limited evidence
Cohort	1	1	0		0	0	
Case Control^^	1	0	1		0	1	
Cross-sectional	0	0	0		0	0	
*Knee pain*				Evidence			Evidence
Cohort	5	4	1		3	0	
Case Control^^	2	1	1		1	1	
Cross-sectional	6	4	2		2	1	
*Foot pain*				Evidence			Limited evidence
Cohort	5	5	0		1	0	
Case Control^^	1	0	1		0	0	
Cross-sectional	2	2	0		0	1	

^ These studies reported anthropometric measures of adiposity, such as waist circumference, waist-hip ratio, and hip circumference, to measure central adiposity.

^^ All case control studies were cross-sectional in design.

# These studies used direct measures of adiposity, such as fat mass and percentage total body fat, to measure total body adiposity.

* A study by Yip and colleagues found an inverse relationship between central adiposity and low back pain (included here).

** A study by Ojoawo et al reported a relationship for hip circumference, but not waist circumference or waist-hip ratio, and low back pain (included here).

**Table 4:** Summary of evidence from cohort studies examining the longitudinal relationship between adiposity and the presence of pain, incident pain and progression of pain.

Region and adiposity measurement	Presence of pain No of cohort studies		Summary of Evidence	Incident pain No of cohort studies		Summary of Evidence	Progression of pain No of cohort studies		Summary of Evidence
	Association	No association		Association	No association		Association	No association	
***Low back pain***			Evidence			Conflicting evidence			Limited evidence
Anthropometric	4	0		1	1		1	0	
Direct	3	0		1	1		0	0	
Both	5 *	0		2	1*		1	0	
***Hip pain***			Limited evidence			No evidence			No evidence
Anthropometric	0	0		0	0		0	0	
Direct	1	0		0	0		0	0	
Both	1	0		0	0		0	0	
***Knee pain***			Evidence			Limited evidence			Limited evidence
Anthropometric	2	0		0	0		2	0	
Direct	2	0		0	1		1	0	
Both	3*	0		0	1		2 *^¥^	0	
***Foot pain***			Evidence			Evidence			Evidence
Anthropometric	0	0		1	0		1	0	
Direct	2	0		2	1		2	0	
Both	2	0		3	1		2*	0	

*The same study provided results relating to anthropometric and direct measures of adiposity.

^¥^ One study examined trajectories of pain, while the other study reported an increase in pain.

### Relationship between adiposity and hip pain

#### Anthropometric fat measures

One case control study found no significant difference in waist circumference, hip circumference and waist-hip ratio between individuals with greater trochanteric pain and controls [102] (Table 5).

**Table 5:** Results of the studies investigating the relationship between adiposity and hip and knee pain.

**Hip pain**				
Case control studies				
*Anthropometric fat measurement*				
Fearon (2012) [102]	Participants diagnosed with trochanteric pain	NA	*Control vs trochanteric pain (WC)* Mean (95% CI)– 83.0cm (78.9–87.1) vs 88.4cm (82.9–93.9), p = 0.42 *Control vs trochanteric pain (HC)* *Mean (95% CI)* 102.8cm (99.4–106.2) vs 109.1cm (104.9–113.2), p = 0.09 *Control vs trochanteric pain (WHR)* Mean (95% CI) 0.81 (0.783–0.837) vs 0.79 (0.75–0.83), p = 0.884	Those with trochanter pain did not have significantly larger WC, HC or WHR compared to controls.
Cohort studies				
*Direct fat measurement*				
Pan (2017) [94]	Responded yes to the presence of hip pain.	Age, sex, height, smoking history, physical activity, emotional problems, education level and employment	**5 year follow-up:** **Presence of pain** (fat mass) OR 1.38 (1.13–1.70)	Hip pain was significantly associated with high fat mass.
			**Presence of pain** (FMI) OR 1.42 (1.13–1.79)	
**Knee pain**				
Cross-sectional studies				
*Anthropometric fat measurement*				
Lee (2016) [125]	Pain intensity was reported on a scale of 1–10. Pain was categorised as mild (1–3), moderate (4–6) or severe (7–10).	NA	**Knee pain vs no knee pain** (WC) Mean (SD) = 85.92 (0.50) vs 85.14 (0.34), p = 0.19	Participants with knee pain did not have significantly higher WC compared to participants with no knee pain.
Frilander (2016) [124]	Responded yes to “Have you during the previous 30 days had pain, ache or motion soreness?”	No adjustments	**Presence of pain** WC (continuous) OR 1.15 (0.99–1.33) **Presence of pain** WC (<94cm ref) 94–101.9cm: OR 1.16 (0.86–1.55) ≥101.9cm: OR 1.38 (1.04–1.82)	Knee pain was significantly associated with WC > 101.9cm among men who served in the Finnish military.
Muramoto (2014) [132]	Pain intensity of ≥ 1 on the VAS.	Age	WC: r = 0.2, p<0.005 HC: r = 0.2, p<0.01 WHR: r = 0.2, p<0.01 Multivariate analyses: WC: significant association reported. Data not provided. p<0.01.	Central obesity was associated with knee pain intensity.
*Direct fat measurement*				
Alfieri (2017) [128]	WOMAC, score of ≥ 1 scale of 0–100	NA	**Adequate adiposity vs excessive adiposity** Mean (SD) = 53.6 (25.6) vs 59.9 (16.8), p<0.05	Participants with excessive adiposity (in accordance with the American College of Sports Medicine (ACSM) recommendation) had a higher pain score on the WOMAC than those with adequate adiposity.
Lee (2016) [125]	Pain intensity was reported on a scale of 1–10. Pain was categorised as mild (1–3), moderate (4–6) and severe (7–10).	Age, sex, physical activity, BMI	**Pain intensity; Severe knee pain (≥7)** Leg to whole body fat mass: OR 1.01 (0.98–1.05)	Fat mass in the leg, relative to whole body, was not correlated with knee symptoms.
Ozer Kaya, (2014) [120]	Pain was graded on the VAS, 0-100mm. Scores of ≥70 were excluded.	NA	**Knee pain vs no knee pain** (body fat percentage) Mean (SD) = 39.29 (7.86) vs 38.13 (7.67), p>0.05 **Knee pain vs no knee pain** (fat mass, kg) Mean (SD) = 30.46 (11.77) vs 28.64 (9.59), p>0.05	There were no significant differences in body fat percentage or fat mass between knee pain and non-knee pain subjects.
Scott (2012) [129]	Responded yes to the question “Do you have pain at any of these sites today?” (for knee pain).	NA	**Males** **Knee pain vs no knee pain** (fat mass %) % (SD) = 28.0 (5.2) vs 27.2 (4.4), p = 0.073	Percentage fat mass was significantly higher in those with knee pain among females, but not males.
			**Females** **Knee pain vs no knee pain** (fat mass %) % (SD) = 40.1 (5.5) vs 39.0 (5.0), p = 0.046	
Case-control studies				
*Anthropometric fat measurement*				
Li (2016) [110]	Pain intensity score ≥1 on the VAS	NA	**Pain intensity** Knee OA vs controls 6.82+/-1.07, 5.93+/-0.88, p = 0.005	A greater WC was associated with increased pain intensity.
Sutbeyaz (2007) [109]	Participants had knee pain most days of the month	NA	**Pain most days vs no pain** (WHR) Mean (SD) = 0.89 (0.08) vs 0.90 (0.08), p = 0.80	WHR was not found to be significantly associated with the presence of knee pain.
*Direct fat measurement*				
Sutbeyaz (2007) [109]	Participants had knee pain most days of the month	NA	**Pain most days vs no pain** (fat mass, kg) Mean (SD) = 29.40 (7.16) vs 33.60 (7.52), p = 0.06	Total fat mass measured by skin fold was not found to be significantly associated with the presence of knee pain.
Cohort studies				
*Anthropometric fat measurement*				
Pan (2020) [101]	Minimal pain’ group: consistently low level of pain Mild pain’ group: a mild level of pain consistent throughout the follow-up. Moderate pain: relatively high level of pain consistent throughout the follow-up.	Age, sex, physical activity, smoking history, unemployment, education level and radiographic knee osteoarthritis.	**10.7 year follow-up:** **Mild vs minimal pain trajectory (WC):** RR: 1.68 (1.25, 2.25) **Moderate vs minimal pain trajectory (WC):** RR: 3.19 (2.12, 4.80)	Central obesity increased risk of ‘Mild pain’ and ‘Moderate pain’ rather than ‘Minimal pain’.
Jin (2016) [89]	Knee pain was defined as a score of ≥1 on a scale of 0–10. Consistent pain was assessed as any pain at baseline and follow up. Fluctuating pain was assessed as pain in any one or two time-points.	Age, gender, height, radiographic OA	**Average 5.1 year follow-up:** **Consistent pain vs no pain** (WHR) RR 1.25 (0.98–1.59) **Fluctuating pain vs no pain** (WHR) RR 1.46 (1.18–1.80)	WHR and WC was found to be a significant predictor of increasing knee pain and more consistently associated with non-weight bearing knee pain. Similarly WHR and WC were significantly associated with an increase in knee pain, however no significant relationship was found with total knee pain.
			**Consistent pain vs no pain** (WC) RR 1.46 (1.18–1.80) **Fluctuating pain vs no pain** (WC) RR 1.55 (1.27–1.89)	
			**Average 5.1 year follow-up:** **Increase in knee pain** (WC) OR 1.37 (1.18–1.59) **Total knee pain** (WC) OR 1.38 (0.97–1.80)	
			**Increase in knee pain** (WHR) OR 1.23 (1.03–1.47) **Total knee pain** (WHR) OR 1.36 (0.83–1.90)	
Batsis (2014) [91]	Pain as assessed on the WOMAC- 5 point Likert scale.	Age, sex, education level, race, cohort type (incident, progression and control), Charlson co-morbidity score, current smoking status, baseline scores where available	**6 year follow-up:** **Right knee pain intensity** (WC, quartiles) Mean (SD) = 8.4 (11.9) vs 10.8 (13.4) vs 12.3 (14.3) vs 14.2 (15.3): ANOVA, p<0.01 **Left knee pain intensity** (WC, quartiles) Mean (SD) = 7.8 (12.6) vs 10.8 (14.6) vs 12.0 (15.3) vs 13.8 (16.1): ANOVA, p<0.01	WOMAC scores were significantly higher in the higher quartile WC groups compared to the lower quartiles.
*Direct fat measurement*				
Pan (2017) [94]	Yes response to presence of pain	Age, sex, height, smoking history, physical activity, emotional problems, education level and employment	**5 year follow-up:** **Presence of pain** (fat mass) OR 1.99 (1.59–2.49)	Knee pain was significantly associated with fat mass and FMI in an older cohort.
			**Presence of pain** (FMI) OR 2.06 (1.60–2.64)	
Jin (2016) [89]	Knee pain was defined as a score of ≥1 on a scale of 0–10. Consistent pain was assessed as any pain at baseline and follow up. Fluctuating pain was assessed as pain in any one or two time-points.	Age, gender, height, radiographic OA	**Average 5.1 year follow-up:** **Consistent pain vs no pain** (fat mass) RR 1.89 (1.43–2.51) **Fluctuating pain vs no pain** (fat mass) RR 1.78 (1.41–2.25)	Body fat mass was found to be a significant predictor of increasing knee pain and more consistently associated with non-weight bearing knee pain.
			**Increase in knee pain** (fat mass) OR 1.36 (1.20–1.55) **Total knee pain** (fat mass) OR 1.17 (0.76–1.59)	
Barber (2012) [86]	Pain was assessed on a 0–100 scale. Scores of 100 represented no pain.	NA	**2 year follow-up:** **Incident patellofemoral pain vs no patellofemoral pain** Mean body fat % (95% CI): 22.2 (19.4–24.9) vs 22.9 (21.8–24.1), p>0.05	No significant difference in body fat percentage was found between middle school female basketball players who developed patellofemoral pain and those who did not.

FMI = fat mass index, HC = hip circumference, NA = not available, OR = odds ratio, RR = relative risk, WC = waist circumference, WOMAC = Western Ontario and McMaster Universities Arthritis Index, WHR = waist-hip ratio.

#### Direct fat measures

*Body fat mass*. A single cohort study found greater body fat mass was associated with the presence of hip pain among older individuals [94].

Overall there was limited evidence for an association between adiposity and hip pain based on two studies with conflicting results (Table 3). There was limited evidence to suggest central adiposity is not a risk factor for hip pain (one case-control study) and limited or no evidence that there is a longitudinal relationship between adiposity and the presence (one cohort study), incidence (no studies) or progression of hip pain (no studies) (Table 4).

### Relationship between adiposity and knee pain

#### Anthropometric fat measures

*Waist circumference*. Seven studies, including three cross-sectional, one case-control and three cohort studies, examined the association between waist circumference and knee pain (Table 5). While one cross-sectional study found a significant difference in knee pain between those with a waist circumference <94cm and those with waist circumference ≥101.9cm [124] and a second cross-sectional study found an association between waist circumference and knee pain intensity [132], a third cross sectional study found no differences in waist circumference between those with and without knee pain [125]. Moreover, a case-control study found that a greater waist circumference was associated with increased pain intensity [110]. Of the three cohort studies, one reported a significant association between waist circumference and consistent and fluctuating knee pain [89], and the other two found a significant relationship between waist circumference and knee pain intensity [91,101].

*Waist-hip ratio*. One cross-sectional study and one case-control study found significant associations between waist-hip ratio and pain intensity [132] and the presence of knee pain on most days [109], while a cohort study reported an association between waist-hip ratio and fluctuating knee pain, but not consistent knee pain [89].

#### Direct fat measures

*Body fat mass*. Four studies examined the relationship between body fat mass and knee pain. Two cross sectional studies found no significant association between fat mass and knee pain [120,125], while one case-control study found no association between fat mass and presence of knee pain on most days [109] (Table 5). The single cohort study found greater body fat mass was associated with the presence of knee pain among older individuals [94].

*Body fat percentage*. Three cross sectional and two cohort studies examined the association between body fat percentage and knee pain. Two cross sectional studies found an association between body fat percentage and knee pain [128], however one found this association only in females [129], while the remaining cross-sectional study found no association between body fat percentage and knee pain [120]. Of the two cohort studies, one study found an association between body fat percentage and consistent and fluctuating knee pain [89], while the other found no association between body fat percentage and incident patellofemoral pain [86].

#### Summary of the evidence

Overall there was evidence from nine of the 13 identified studies for an association between adiposity and knee pain (Table 3). There was evidence to indicate that central adiposity is a risk factor for knee pain (six of 8 studies) and there is a longitudinal relationship between adiposity and the presence of knee pain (three of three cohort studies) (Table 4). However, there was limited evidence for a relationship between adiposity and incident and increasing knee pain with a limited number of cohort studies identified in each case.

### Relationship between adiposity and foot pain

#### Anthropometric fat measures

*Waist circumference*. One cohort study found that individuals with a larger waist circumference were at greater risk of incident and increasing foot pain [98] (Table 6).

**Table 6:** Results of the studies investigating the relationship between adiposity and foot pain.

**Foot pain**				
Cross-sectional studies				
*Anthropometric fat measurement*				
Butterworth (2016) [126]	Foot pain was defined as a score of ≥1 with minimum and maximum scores ranging from 0–19	Age, depression, mobility and education.	**Foot pain vs no foot pain** (WHR) OR 1.02 (0.99–1.06)	Presence of foot pain was not associated with WHR.
*Direct fat measurement*				
Butterworth (2016) [126]	Foot pain was defined as a score of ≥1 with minimum and maximum scores ranging from 0–19	Age, depression, mobility, education, residual of weight on fat mass or BMI on FMI respectively.	**Foot pain vs no foot pain** (total fat mass) OR 1.02 (1.003–1.05) **Foot pain vs no foot pain** (FMI) OR 1.08 (1.01–1.15)	In men, fat mass, but not WHR, was associated with having foot pain.
Tanamas (2012) [119]	Foot pain was classified as having current foot pain and pain in the last month as well as a score of ≥1 with minimum and maximum scores ranging from 0–19	Total fat mass: age, sex, skeletal muscle mass FMI: age, sex, FFMI All others: age, sex	**Foot pain vs no foot pain** (total fat mass) OR 1.05 (1.02–1.09)	The effect of obesity on foot pain was related to an increase in adiposity, particularly in the android distribution of fat. In contrast, the gynoid distribution of fat was found to have a beneficial effect.
			**Foot pain vs no foot pain** (FMI) OR 1.16 (1.06–1.28)	
			**Foot pain vs no foot pain** (total body fat %) OR 1.10 (1.05–1.14)	
			**Foot pain vs no foot pain** (android/total body fat ratio) OR 1.42 (1.11–1.83)	
			**Foot pain vs no foot pain** (gynoid/total body fat ratio) OR 0.83 (0.73–0.93)	
			**Foot pain vs no foot pain** (trunk/total body fat ratio) OR 1.05 (0.98–1.12)	
Case control studies				
*Direct fat measurement*				
Walsh (2017) [54]	Score of ≥ 1 on the VAS	N/A	**Foot pain group vs control group** (fat mass, kg) 33.1 vs 31.5 p = 0.578	There was no significant difference in total fat mass between the foot pain and control groups.
Cohort studies				
*Anthropometric fat measurement*				
Laslett (2018) [98]	Pain (yes/no)	Age, gender	**5 year follow-up:** **Incident foot pain** (WC) RR 1.22 (1.01–1.49) **Change in foot pain (**WC) RR 1.26 (1.19–1.34)	Individuals with a greater WC were at higher risk of incident foot pain and an increase in foot pain. However, individuals with greater WHR were only at risk of an increase in foot pain.
			**Incident foot pain** (WHR) RR 1.23 (0.95–1.61) **Change in foot pain (**WHR) RR 1.27 (1.16–1.39)	
*Direct fat measurement*				
Laslett (2018) [98]	Pain (yes/no)	Age, gender	**5 year follow-up:** **Incident foot pain** Total fat mass RR 0.92 (0.71–1.19) FMI RR 1.16 (0.93–1.46)	Individuals with greater total fat mass and FMI were at higher risk of increases in foot pain. However, greater total fat mass and FMI were not associated with incident foot pain.
			**5 year follow-up:** **Change in foot pain** Total fat mass RR 1.28 (1.18–1.40) FMI RR 1.21 (1.18–1.24)	
Walsh (2018) [99]	Assessed by Manchester-Oxford foot questionnaire and converted to 100 scale	Age, gender, depression, treatment group	**4–20 year follow-up:** **Change in foot pain** (FMI) β coefficient 1.5 (0.2–2.8)	FMI was a predictor of change in foot pain.
Pan (2017) [94]	Presence of pain (yes/no)	Age, sex, height, smoking history, physical activity, emotional problems, education level and employment	**5 year follow-up:** **Presence of pain** (fat mass) OR 1.87 (1.51–2.32)	Foot pain was associated with fat mass and FMI in an older cohort.
			**Presence of pain** (FMI) OR 1.99 (1.57–2.53)	
Walsh (2016) [90]	Prevalent (presence of) pain defined as responding yes to “On most days, do you have pain, aching, or stiffness in either of your feet?” Future foot pain was defined as responding yes to “Over the past month, have you had pain, aching, or stiffness in either of your feet on most days?”	BMI, FFMI, WHR, age, IL-6 level, TNF level	**3–4 year follow-up:** **Presence of foot pain vs no foot pain** (FMI) OR 1.08 (1.04–1.12)	FMI was positively associated with both the presence of foot pain and future foot pain.
			**3-4year follow-up:** **Future foot pain vs no foot pain** (FMI) OR 1.06 (1.02–1.11)	
Butterworth (2013) [85]	Foot pain was defined as a score of ≥1 with minimum and maximum scores ranging from 0–19	Age, gender, mental component summary, total fat-free mass/FFMI respectively	**3 year follow-up** **Incident foot pain vs no foot pain** (total fat mass) OR 1.11 (1.03–1.20)	Total fat mass was found to be a predictor of incident foot pain.
			**Incident foot pain vs no foot pain** (FMI) OR 1.28 (1.04–1.57)	

BMI = body mass index, FFMI = fat free mass index, FMI = fat mass index, OR = odds ratio, WHR = waist-hip ratio, WHtR = Waist-height ratio.

*Waist-hip ratio*. One cross-sectional study, which examined the association between waist-hip ratio and foot pain, found no significant association [126].

#### Direct fat measures

*Body fat mass*. Seven studies, including two cross-sectional, one case control and five cohort studies, examined the association between fat mass measures and foot pain (Table 6). Both cross -sectional studies found significant associations between fat mass and foot pain [119,126], while the case-control study did not find any significant difference between total fat mass in individuals with foot pain compared to those without [106]. Five cohort studies found significant positive relationships between direct fat measures and foot pain, with two studies reporting an association with the presence of foot pain [90,94], three studies finding a relationship with incident foot pain [85,90,99] and two studies reporting an association with progression of foot pain [98,99]. While Laslett and colleagues reported a relationship between fat measures and increasing foot pain, no association was found for incident foot pain [98].

#### Summary of the evidence

Overall there was evidence from seven of the eight identified studies for an association between adiposity measures and foot pain (Table 3). While there was limited evidence for a relationship between central adiposity and foot pain (two conflicting studies), there was evidence for a longitudinal relationship between adiposity and the presence of pain (two of two studies), incident foot pain (three of four studies) and progression of foot pain from 6 months to 5 years (two of two studies) (Table 4).

## Discussion

This systematic review found that both body fat and its central distribution are associated with musculoskeletal pain. There was evidence of a relationship between central adiposity and low back and knee pain, but limited or conflicting evidence for hip and foot pain. There was also evidence of a longitudinal relationship between adiposity and the presence of low back, knee and foot pain, as well as both incident and increasing foot pain. Taken together, these findings further our understanding of the mechanisms underlying obesity-related musculoskeletal pain and highlight adiposity as a potential therapeutic target in the management of back and lower leg pain.

This systematic review is the first to examine the relationship between fat distribution and musculoskeletal pain. We found evidence that central adiposity, defined as the accumulation of extra subcutaneous and visceral fat concentrated just above or around the waistline, was associated with pain in the lower back and knee. This was based on evidence of a significant, positive association in 16 of the 22 studies of low back pain, including five of six cohort studies, and 6 of 8 studies of knee pain, including 3 of 3 cohort studies. This finding is consistent with evidence that central adiposity is associated with a greater risk of major public health conditions, such as cardiovascular disease and diabetes, which are associated with huge socioeconomic burdens globally [11]. Given visceral fat associated with central adiposity is an important correlate of metabolic disturbances [139], and the cells in central, visceral fat have a much higher turnover than subcutaneous fat cells in other regions of the body [140], central adiposity may be an important way to target obesity. For instance, management strategies targeted to enhance weight loss around the abdominal region may be particularly beneficial. Overall, the evidence for an association between central adiposity and low back and knee pain indicates that it is not just extra body fat that contributes to poor health and chronic pain, but also the distribution of the fat.

This review found evidence of a longitudinal relationship between adiposity and the presence of low back, knee and foot pain, as well as incident and increasing foot pain. The findings suggest that increased adiposity can lead to back and lower limb pain in the future, and in the case of foot pain, the development or increasing intensity of pain. Our results, which take into account 17 cohort studies, build on the conclusions of a previous review of seven cohort studies [10], which suggested that such associations may exist, but was limited by a lack of high quality studies. Our results highlight the need for high quality clinical trials to examine the efficacy of approaches that target weight loss, be it through physical activity, diet and/or medical options, in the management of back and lower limb pain in overweight and obese individuals. They also suggest that investigating the efficacy of the targeted interventions, such as exercise programs that focus on reducing adipose tissue and nutrition plans that optimize health but minimize fat intake. Moreover, given current evidence collectively indicates that musculoskeletal pain has an important systemic inflammatory component, there is an exciting opportunity to examine the efficacy of pharmaceutical and complementary medicines as potential treatment targets to reduce inflammation in individuals with musculoskeletal pain with a specific overweight/obese profile.

Moreover, evidence from this review informs our knowledge of the mechanisms that underlie obesity-related musculoskeletal pain. Several mechanisms, including increased physical loading and systemic metabolic processes, have been proposed to explain the role of obesity in musculoskeletal pain. In overweight or obese individuals, excess adipose tissue may result in increased load on a region and subsequently, altered posture and abnormal movement patterns resulting in pain and disability. There is also growing evidence to support systemic metabolic processes, with evidence that adiposity is associated with pain in non-weight-bearing regions, such as the hand [94]. Adipose tissue is metabolically active, releasing a multitude of pro-inflammatory cytokines and adipokines, which may potentiate inflammatory changes in a region resulting in pain [141]. Moreover, inflammation has been shown to alter the excitation thresholds and responses to stimuli of peripheral nerves, subsequently leading to peripheral and central sensitisation [142,143]. Our findings provide evidence that both mechanical and metabolic mechanisms may be at play in lower back, knee and foot pain, with the potential for total body fat and central obesity to load these regions and increased visceral and subcutaneous fat to alter metabolic processes. However, preliminary evidence from studies that reported adiposity to be associated with pain in non-weight bearing regions, such as the neck and hand, suggest that future research examining these regions may further our understanding of the pathogenesis of obesity-related musculoskeletal pain.

This systematic review has several important strengths, including conducting a comprehensive, systematic search of the literature based on six electronic databases, performing a risk of bias assessment of studies using the Cochrane risk of bias assessment, and conducting a best evidence synthesis to summarise the strength of the available evidence. Moreover, this review is novel, as it is the first to provide evidence of the role of central adiposity in site-specific musculoskeletal pain, as well as an updated summary of the evidence examining the longitudinal association between adiposity and back and lower limb pain. While this review was not registered a priori with an international prospective register, we have provided a detailed description of our review methodology from development of our search strategy to the assigning of levels of evidence and documented any changes in our initial methodology in this publication. Furthermore, while the review was limited by the paucity of high quality cohort studies, as well as significant heterogeneity in the identified studies, which meant a meta-analysis could not be performed, we used established levels of evidence to summarise the data for each musculoskeletal pain region

This systematic review found that both body fat and its distribution are associated with site-specific musculoskeletal pain. There was evidence of a positive relationship between central adiposity and low back and knee pain and a longitudinal association between adiposity and the presence of back, knee and foot pain, as well as incident and worsening foot pain. These findings are not only important in understanding the mechanisms which underlie chronic, musculoskeletal pain, but in the development of innovative treatment approaches for these debilitating conditions.

## Supporting information

S1 Checklist PRISMA checklist. (DOC)

S1 Text Medline database search strategy. (DOCX)

## References

[1] GBD 2016 Disease and Injury Incidence and Prevalence Collaborators. Global, regional, and national incidence, prevalence, and years lived with disability for 328 diseases and injuries for 195 countries, 1990–2016: a systematic analysis for the Global Burden of Disease Study 2016. Lancet. 2017; 390(10100): 1211–59. doi: 10.1016/S0140-6736(17)32154-2 28919117 PMC5605509

[2] Briggs AM, Cross MJ, Hoy DG, Sanchez-Riera L, Blyth FM, Woolf AD, et al. Musculoskeletal Health Conditions Represent a Global Threat to Healthy Aging: A Report for the 2015 World Health Organization World Report on Ageing and Health. The Gerontologist. 2016; 56 Suppl 2: S243–55. doi: 10.1093/geront/gnw002 26994264

[3] Gregg E, Shaw J. Global health effects of overweight and obesity. N Engl J Med. 2017; 377(1): 80–1. doi: 10.1056/NEJMe1706095 28604226

[4] Shiri R, Karppinen J, Leino-Arjas P, Solovieva S, Viikari-Juntura E. The Association Between Obesity and Low Back Pain: A Meta-Analysis. American Journal of Epidemiology. 2010; 171(2): 135–54. doi: 10.1093/aje/kwp356 20007994

[5] Butterworth P, Landorf K, Smith S, Menz H. The association between body mass index and musculoskeletal foot disorders: a systematic review. Obes Rev. 2012; 13(7): 630–42. doi: 10.1111/j.1467-789X.2012.00996.x 22498495

[6] Urquhart DM, Berry P, Wluka AE, Strauss BJ, Wang Y, Proietto J, et al. 2011 Young Investigator Award winner: Increased fat mass is associated with high levels of low back pain intensity and disability. Spine (Phila Pa 1976). 2011; 36(16): 1320–5. doi: 10.1097/BRS.0b013e3181f9fb66 21270692

[7] Chou L, Brady SR, Urquhart DM, Teichtahl AJ, Cicuttini FM, Pasco JA, et al. The association between obesity and low back pain and disability is affected by mood disorders: A population-based, cross-sectional study of men. Medicine. 2016; 95(15): e3367. doi: 10.1097/MD.0000000000003367 27082599 PMC4839843

[8] Coppack SW. Pro-inflammatory cytokines and adipose tissue. The Proceedings of the Nutrition Society. 2001; 60(3): 349–56. doi: 10.1079/pns2001110 11681809

[9] Willard F. 8—Neuroendocrine—immune network, nociceptive stress and the general adaptive response. In: Everett T, Dennis M, Ricketts E, editors. Physiotherapy in Mental Health: Butterworth-Heinemann; 1995. p. 102–26.

[10] Walsh TP, Arnold JB, Evans AM, Yaxley A, Damarell RA, Shanahan EM. The association between body fat and musculoskeletal pain: a systematic review and meta-analysis. BMC Musculoskelet Disord. 2018; 19(1): 233. doi: 10.1186/s12891-018-2137-0 30021590 PMC6052598

[11] World Health Organization. Waist Circumference and Waist-hip Ratio: Report of a WHO Expert Consultation. Geneva; 2011.

[12] Moher D, Liberati A, Tetzlaff J, Altman DG. Preferred Reporting Items for Systematic Reviews and Meta-Analyses: The PRISMA Statement. Annals of Internal Medicine. 2009; 151(4): 264–9. doi: 10.7326/0003-4819-151-4-200908180-00135 19622511

[13] Duren DL, Sherwood RJ, Czerwinski SA, Lee M, Choh AC, Siervogel RM, et al. Body composition methods: comparisons and interpretation. J Diabetes Sci Technol. 2008; 2(6): 1139–46. doi: 10.1177/193229680800200623 19885303 PMC2769821

[14] Higgins JPT, Altman DG, Gøtzsche PC, Jüni P, Moher D, Oxman AD, et al. The Cochrane Collaboration’s tool for assessing risk of bias in randomised trials. BMJ. 2011; 343. doi: 10.1136/bmj.d5928 22008217 PMC3196245

[15] Lievense AM, Bierma-Zeinstra SM, Verhagen AP, van Baar ME, Verhaar JA, Koes BW. Influence of obesity on the development of osteoarthritis of the hip: a systematic review. Rheumatology (Oxford, England). 2002; 41(10): 1155–62. doi: 10.1093/rheumatology/41.10.1155 12364636

[16] Urquhart D, Zheng Y, Cheng A, Rosenfeld J, Chan P, Liew S, et al. Could low grade bacterial infection contribute to low back pain? A systematic review. BMC Med. 2015; 13: 13. doi: 10.1186/s12916-015-0267-x 25609421 PMC4320560

[17] MacLellan G, Dunlevy C, E OM, Blake C, Breen C, Gaynor K, et al. Musculoskeletal pain profile of obese individuals attending a multidisciplinary weight management service. Obesity Facts. 2017; 10 (Supplement 1): 193. doi: 10.1097/j.pain.0000000000000918 28383311

[18] Dufour AB, Losina E, Menz HB, LaValley MP, Hannan MT. Obesity, foot pain and foot disorders in older men and women. Obesity Research and Clinical Practice. 2017; 11(4): 445–53. doi: 10.1016/j.orcp.2016.11.001 27887922 PMC5440224

[19] Najafipour H, Sadeghigoghari M, Kordestani Z, Tahami AN, Ghavipisheh M. Prevalence of the musculoskeletal pain syndrome and its associated factors in people between 15 and 80 years in kerman: A population-based study on 1700 individuals. Iranian Red Crescent Medical Journal. 2017; 19(4).

[20] Okamoto CS, Dunn AS, Green BN, Formolo LR, Chicoine D. Correlation of Body Composition and Low Back Pain Severity in a Cross-Section of US Veterans. Journal of Manipulative and Physiological Therapeutics. 2017; 40(5): 358–64. doi: 10.1016/j.jmpt.2017.03.003 28554432

[21] Peng T, Perez A, Pettee Gabriel K. The Association Among Overweight, Obesity, and Low Back Pain in U.S. Adults: A Cross-Sectional Study of the 2015 National Health Interview Survey. Journal of Manipulative and Physiological Therapeutics. 2018. 10.1016/j.jmpt.2017.10.005 29459122

[22] Higgins D, Buta E, Heapy A, Driscoll M, Kerns R, Masheb R, et al. The relationship among BMI, pain intensity, and musculoskeletal diagnoses. Journal of Pain. 2016; 1): S28.

[23] DePalma MJ, Ketchum JM, Kouchouk A, Powell D, Ruchala MD. Multivariate analyses of age, gender, and BMI on the source of low back pain. PM and R. 2010; 1): S62.

[24] Segar AH, Urban JPG, Fairbank JCT, Judge A. The association between body mass index (BMI) and back or leg pain in patients with spinal conditions results from the genodisc study. Spine. 2016; 41(20): E1237–E43. doi: 10.1097/BRS.0000000000001606 27760064

[25] Aoyagi K, Ross PD, Okano K, Hayashi T, Moji K, Kusano Y, et al. Association of body mass index with joint pain among community-dwelling women in Japan. Aging—Clinical and Experimental Research. 2002; 14(5): 378–81. doi: 10.1007/BF03324465 12602572

[26] Brooks C, Siegler JC, Cheema BS, Marshall PWM. No relationship between body mass index and changes in pain and disability after exercise rehabilitation for patients with mild to moderate chronic low back pain. Spine. 2013; 38(25): 2190–5. doi: 10.1097/BRS.0000000000000002 24296481

[27] Hagen K, Heuch I, Nygaard O, Zwart JA. The impact of body mass index on the prevalence of low back pain: The HUNT study. Spine. 2010; 35(7): 764–8. doi: 10.1097/BRS.0b013e3181ba1531 20228714

[28] Macfarlane GJ, De silva V, Jones GT. The relationship between body mass index across the life course and knee pain in adulthood: Results from the 1958 birth cohort study. Rheumatology. 2011; 50(12): 2251–6. doi: 10.1093/rheumatology/ker276 21984765

[29] Ozkaya DB, Onsun N, Topukcu B, Su O, Bahali AG, Dizman D, et al. The relationship between body mass index, waist circumference and psoriatic arthritis in the Turkish population. Postepy Dermatologii i Alergologii. 2016; 33(3): 219–23. doi: 10.5114/ada.2016.60615 27512358 PMC4969418

[30] Sajan CK, Malik C, Srikrishnan MT. The association between body mass index and low back pain on the quality of life. PM and R. 2011; 1): S217.

[31] Singh JA, Gabriel SE, Lewallen DG. Higher Body Mass Index Is Not Associated With Worse Pain Outcomes After Primary or Revision Total Knee Arthroplasty. Journal of Arthroplasty. 2011; 26(3): 366–74.e1. 10.1016/j.arth.2010.02.006 PMC2930933 20413245

[32] Cadish LA, Hacker MR, Dodge LE, Dramitinos P, Hota LS, Elkadry EA. Association of body mass index with hip and thigh pain following transobturator midurethral sling placement. American Journal of Obstetrics and Gynecology. 2010; 203(5): 508.e1–.e5. doi: 10.1016/j.ajog.2010.07.023 20728070

[33] Brady S, Hussain SM, Brown W, Heritier S, Urquhart D, Wang Y, et al. The course and contributors to back pain in middle-aged women over nine years: Data from the australian longitudinal study on women’s health. Internal Medicine Journal. 2018; 48 (Supplement 4): 10. 10.1097/BRS.0000000000002702 29794589

[34] Cooper L, Ells L, Ryan C, Martin D. Perceptions of adults with overweight/obesity and chronic musculoskeletal pain: An interpretative phenomenological analysis. Journal of clinical nursing. 2018; 27(5–6): e776–e86. doi: 10.1111/jocn.14178 29148620

[35] Li J, Chen J, Qin Q, Zhao D, Dong B, Ren Q, et al. Chronic pain and its association with obesity among older adults in China. Archives of Gerontology & Geriatrics. 2018; 76: 12–8. doi: 10.1016/j.archger.2018.01.009 29427812

[36] Su CA, Kusin DJ, Li SQ, Ahn UM, Ahn NU. The Association between Body Mass Index and the Prevalence, Severity, and Frequency of Low Back Pain. Spine. 2018; 43(12): 848–52. doi: 10.1097/BRS.0000000000002601 29462069

[37] Angst F, Angst J, Ajdacic-Gross V, Aeschlimann A, Rossler W. Epidemiology of Back Pain in Young and Middle-Aged Adults: A Longitudinal Population Cohort Survey From Age 27–50 Years. Psychosomatics. 2017. doi: 10.1016/j.psym.2017.05.004 28867433

[38] Baek SR, Lim JY, Lim JY, Park JH, Lee JJ, Lee SB, et al. Prevalence of musculoskeletal pain in an elderly Korean population: Results from the Korean Longitudinal Study on Health and Aging (KLoSHA). Archives of Gerontology and Geriatrics. 2010; 51(3): e46–e51. doi: 10.1016/j.archger.2009.11.011 20005585

[39] Borg JH, Westerstahl M, Lundell S, Madison G, Aasa U. Longitudinal study exploring factors associated with neck/shoulder pain at 52 years of age. Journal of Pain Research. 2016; 9: 303–10. doi: 10.2147/JPR.S93845 27307762 PMC4889214

[40] Fernandez-De-Las-Penas C, Alonso-Blanco C, Hernandez-Barrera V, Palacios-Cena D, Jimenez-Garcia R, Carrasco-Garrido P. Has the prevalence of neck pain and low back pain changed over the last 5 years? A population-based national study in Spain. Spine Journal. 2013; 13(9): 1069–76. 10.1016/j.spinee.2013.02.064 23578987

[41] Fernandez-De-Las-Penas C, Hernandez-Barrera V, Alonso-Blanco C, Palacios-Cena D, Carrasco-Garrido P, Jimenez-Sanchez S, et al. Prevalence of neck and low back pain in community-dwelling adults in spain: A population-based national study. Spine. 2011; 36(3): E213–E9. doi: 10.1097/BRS.0b013e3181d952c2 21079541

[42] Gandhi R, Perruccio AV, Rizek R, Dessouki O, Evans HMK, Mahomed NN. Obesity-related adipokines predict patient-reported shoulder pain. Obesity Facts. 2013; 6(6): 536–41. doi: 10.1159/000357230 24335140 PMC5644779

[43] Hellsing AL, Bryngelsson IL. Predictors of musculoskeletal pain in men: A twenty-year follow-up from examination at enlistment. Spine. 2000; 25(23): 3080–6. doi: 10.1097/00007632-200012010-00016 11145820

[44] Honda R, Noborisaka Y, Ishida M, Ishizaki M, Yamada Y. Impact of obesity on musculoskeletal pain and difficulty of daily movements in Japanese middle-aged women. Maturitas. 2002; 42(1): 23–30. doi: 10.1016/s0378-5122(02)00025-7 12020976

[45] Kaaria S, Laaksonen M, Rahkonen O, Lahelma E, Leino-Arjas P. Risk factors of chronic neck pain: A prospective study among middle-aged employees. European Spine Journal. 2012; 21 (5): 1022. doi: 10.1002/j.1532-2149.2011.00065.x 22337254

[46] Mork PJ, Vik KL, Moe B, Lier R, Bardal EM, Nilsen TI. Sleep problems, exercise and obesity and risk of chronic musculoskeletal pain: the Norwegian HUNT study. European Journal of Public Health. 2014; 24(6): 924–9. doi: 10.1093/eurpub/ckt198 24293504

[47] Peltonen M, Lindroos AK, Torgerson JS. Musculoskeletal pain in the obese: A comparison with a general population and long-term changes after conventional and surgical obesity treatment. Pain. 2003; 104(3): 549–57. doi: 10.1016/S0304-3959(03)00091-5 12927627

[48] Tsuritani I, Honda R, Noborisaka Y, Ishida M, Ishizaki M, Yamada Y. Impact of obesity on musculoskeletal pain and difficulty of daily movements in Japanese middle-aged women. Maturitas. 2002; 42(1): 23–30. doi: 10.1016/s0378-5122(02)00025-7 12020976

[49] Brooks JM, Deiches J, Xiaoling X, Batsis JA, Fong C, DiMilia P, et al. Differences in Self-Reported Physical Activity, Exercise Self-Efficacy and Outcome Expectancies, and Health Status by Body Mass Index Groups in People with Chronic Pain. Journal of Rehabilitation. 2018; 84(4): 46–52. 32089565 PMC7034931

[50] Lee SH, Son C, Yeo S, Ha IH. Cross-sectional analysis of self-reported sedentary behaviors and chronic knee pain among South Korean adults over 50 years of age in KNHANES 2013–2015. BMC Public Health. 2019; 19(1): 1375. doi: 10.1186/s12889-019-7653-9 31655569 PMC6815384

[51] Schwarze M, Hauser W, Schmutzer G, Brahler E, Beckmann NA, Schiltenwolf M. Obesity, depression and hip pain. Musculoskeletal Care. 2019; 17(1): 126–32. doi: 10.1002/msc.1380 30623588

[52] Noormohammadpour P, Mansournia MA, Koohpayehzadeh J, Asgari F, Rostami M, Rafei A, et al. Prevalence of chronic neck pain, low back pain, and knee pain and their related factors in community-dwelling adults in Iran: A population-based national study. Clinical Journal of Pain. 2017; 33(2): 181–7. doi: 10.1097/AJP.0000000000000396 27258995

[53] Suri P, Boyko EJ, Smith NL, Jarvik JG, Williams FMK, Jarvik GP, et al. Modifiable risk factors for chronic back pain: insights using the co-twin control design. Spine Journal. 2017; 17(1): 4–14. doi: 10.1016/j.spinee.2016.07.533 27794503 PMC6126929

[54] Walsh TP, Butterworth PA, Urquhart DM, Cicuttini FM, Landorf KB, Wluka AE, et al. Increase in body weight over a two-year period is associated with an increase in midfoot pressure and foot pain. Journal of foot and ankle research. 2017; 10: 31. doi: 10.1186/s13047-017-0214-5 28770005 PMC5526261

[55] Goulston L, D’Angelo S, Sanchez M, Spector T, Hart D, Arden N. Is waist circumference a better predictor of incident symptomatic radiographic knee osteoarthritis, radiographic knee osteoarthritis and knee pain than body mass index over 10 years? Osteoarthritis and Cartilage. 2016; 24: S206–S7.

[56] Yang L, Mu L, Huang K, Zhang T, Mei Z, Zeng W, et al. Abdominal adipose tissue thickness measured using magnetic resonance imaging is associated with lumbar disc degeneration in a Chinese patient population. Oncotarget. 2016; 7(50): 82055–62. doi: 10.18632/oncotarget.13255 27833090 PMC5347673

[57] Ruhdorfer AS, Wirth W, Eckstein F. Decline of thigh muscle cross-sectional areas in chronically painful vs. matched painless knees: Data from the osteoarthritis initiative. Osteoarthritis and Cartilage. 2014; 1): S327–S8. 10.1016/j.joca.2015.04.004 PMC4516618 25887367

[58] Jentzsch T, Geiger J, Slankamenac K, Wanner GA, Simmen HP, Werner CML. Obesity measured by outer abdominal fat may cause facet joint arthritis at the lumbar spine. Swiss Medical Weekly. 2014; 204): 30S. 10.3233/BMR-140495 24968801

[59] Segar A, Urban J, Fairbank J, Judge A. The influence of obesity on back and leg pain in spinal patients: A study of 2,636 patients. Osteoarthritis and Cartilage. 2015; 2): A378–A9.

[60] Arranz L, Canela MA, Rafecas M. Relationship between body mass index, fat mass and lean mass with SF-36 quality of life scores in a group of fibromyalgia patients. Rheumatology International. 2012; 32(11): 3605–11. doi: 10.1007/s00296-011-2250-y 22095395

[61] Jespersen E, Verhagen E, Holst R, Klakk H, Heidemann M, Rexen CT, et al. Total body fat percentage and body mass index and the association with lower extremity injuries in children: a 2.5-year longitudinal study. British journal of sports medicine. 2014; 48(20): 1497–502. doi: 10.1136/bjsports-2013-092790 24273306

[62] Hashem LE, Roffey DM, Alfasi AM, Papineau GD, Wai DC, Phan P, et al. Exploration of the Inter-Relationships Between Obesity, Physical Inactivity, Inflammation, and Low Back Pain. Spine (03622436). 2018; 43(17): 1218–24. 10.1097/BRS.0000000000002582 29419713

[63] Cooper DJ, Scammell BE, Batt ME, Palmer D. Factors associated with pain and osteoarthritis at the hip and knee in Great Britain’s Olympians: a cross-sectional study. British Journal of Sports Medicine. 2018; 52(17): 1101–8. doi: 10.1136/bjsports-2017-098315 29760167

[64] Quittner M, Rantalainen T, Ridgers ND, Trudel G, Sheikh A, Connell D, et al. Intervertebral disc status is associated with vertebral marrow adipose tissue and muscular endurance. European Spine Journal. 2018; 27(8): 1704–11. doi: 10.1007/s00586-018-5567-3 29626268

[65] Resnick B, Hebel JR, Gruber-Baldini AL, Hicks GE, Hochberg MC, Orwig D, et al. The impact of body composition, pain and resilience on physical activity, physical function and physical performance at 2 months post hip fracture. Archives of Gerontology & Geriatrics. 2018; 76: 34–40. doi: 10.1016/j.archger.2018.01.010 29455057 PMC5882522

[66] Schlaeger S, Inhuber S, Rohrmeier A, Dieckmeyer M, Freitag F, Klupp E, et al. Association of paraspinal muscle water-fat MRI-based measurements with isometric strength measurements. European Radiology. 2019; 29(2): 599–608. doi: 10.1007/s00330-018-5631-8 30014202

[67] Goubert D, Meeus M, Willems T, De Pauw R, Coppieters I, Crombez G, et al. The association between back muscle characteristics and pressure pain sensitivity in low back pain patients. Scandinavian Journal of Pain. 2018; 18(2): 281–93. doi: 10.1515/sjpain-2017-0142 29794309

[68] Davison M, Keir P, Maly M, Adachi J, Beattie K. Knee pain intensity is associated with muscle adiposity in the whole thigh and hamstrings of women with knee osteoarthritis. Journal of Rheumatology. 2016; 43 (6): 1149–50.

[69] Davison M, Maly MR, Adachi JD, Beattie KA. Calf muscle adiposity is associated with impaired physical performance in knee OA. Arthritis and Rheumatology Conference: American College of Rheumatology/Association of Rheumatology Health Professionals Annual Scientific Meeting, ACR/ARHP. 2015; 67(SUPPL. 10).

[70] Dannhauer T, Ruhdorfer A, Sattler M, Wirth W, Eckstein F. Cross sectional and longitudinal relationship of thigh adipose tissue with knee pain, radiographic oa status, and structural progression-data from the osteoarthritis initiative. Osteoarthritis and Cartilage. 2014; 1): S331.

[71] Wang J, Han W, Wang X, Pan F, Liu Z, Halliday A, et al. Mass effect and signal intensity alteration in the suprapatellar fat pad: Associations with knee symptoms and structure. Osteoarthritis and Cartilage. 2014; 22(10): 1619–26. doi: 10.1016/j.joca.2014.05.018 24882527

[72] Le Cara EC, Marcus RL, Dempsey AR, Hoffman MD, Hebert JJ. Morphology versus function: The relationship between lumbar multifidus intramuscular adipose tissue and muscle function among patients with low back pain. Archives of Physical Medicine and Rehabilitation. 2014; 95(10): 1846–52. doi: 10.1016/j.apmr.2014.04.019 24814564

[73] Dannhauer T, Ruhdorfer A, Wirth W, Eckstein F. Quantitative relationship of thigh adipose tissue with pain, radiographic status, and progression of knee osteoarthritis: Longitudinal findings from the osteoarthritis initiative. Investigative Radiology. 2015; 50(4): 268–74. doi: 10.1097/RLI.0000000000000113 25419827

[74] Maly MR, Calder KM, MacIntyre NJ, Beattie KA. Relationship of intermuscular fat volume in the thigh with knee extensor strength and physical performance in women at risk of or with knee osteoarthritis. Arthritis Care and Research. 2013; 65(1): 44–52. doi: 10.1002/acr.21868 23044710 PMC3535556

[75] De Almeida AC, Pedroso MG, Aily JB, Goncalves GH, Pastre CM, Mattiello SM. Influence of a periodized circuit training protocol on intermuscular adipose tissue of patients with knee osteoarthritis: Protocol for a randomized controlled trial. BMC Musculoskeletal Disorders. 2018; 19 (1) (no pagination)(421). doi: 10.1186/s12891-018-2325-y 30497420 PMC6267088

[76] Blumel JE, Arteaga E, Mezones-Holguin E, Zuniga MC, Witis S, Vallejo MS, et al. Obesity is associated with a higher prevalence of musculoskeletal pain in middle-aged women. Gynecological Endocrinology. 2017; 33(5): 378–82. doi: 10.1080/09513590.2016.1269741 28084176

[77] Seo YI, Kim HA, Cho NH, Yoo JJ. Relationship between body mass index, fat mass and muscle mass withmusculoskeletal pain in community residents. Arthritis and Rheumatism. 2013; 10): S467.

[78] Mundal I, Grawe RW, Bjorngaard JH, Linaker OM, Fors EA. Prevalence and long-term predictors of persistent chronic widespread pain in the general population in an 11-year prospective study: The HUNT study. BMC Musculoskeletal Disorders. 2014; 15 (1) (no pagination)(213). 24951013 10.1186/1471-2474-15-213 PMC4089927

[79] Park IY, Cho NH, Lim SH, Kim HA. Gender-specific associations between fat mass, metabolic syndrome and musculoskeletal pain in community residents: A three-year longitudinal study. PLoS ONE. 2018; 13 (7) (e0200138). doi: 10.1371/journal.pone.0200138 29985938 PMC6037368

[80] Vehmas T, Shiri R, Luoma K, Viikari-Juntura E. The relations of obesity indicators and early metabolic disturbance with upper extremity pain. Pain medicine (Malden, Mass). 2013; 14(7): 1081–7. doi: 10.1111/pme.12132 23647726

[81] Brady SR, Mamuaya BB, Cicuttini F, Wluka AE, Wang Y, Hussain SM, et al. Body composition is associated with multisite lower body musculoskeletal pain in a community-based study. The journal of pain: official journal of the American Pain Society. 2015; 16(8): 700–6. 10.1016/j.jpain.2015.04.006 25958316

[82] Mikkonen PH, Laitinen J, Remes J, Tammelin T, Taimela S, Kaikkonen K, et al. Association between overweight and low back pain: a population-based prospective cohort study of adolescents. Spine (Phila Pa 1976). 2013; 38(12): 1026–33. doi: 10.1097/BRS.0b013e3182843ac8 23459137

[83] Bjurvald LM, Morinder G, Janson A. Musculoskeletal pain in obese children and adolescents at a specialized pediatric obesity clinic. Obesity Facts. 2018; 11 (Supplement 1): 189.

[84] Deere KC, Clinch J, Holliday K, McBeth J, Crawley EM, Sayers A, et al. Obesity is a risk factor for musculoskeletal pain in adolescents: Findings from a population-based cohort. Pain. 2012; 153(9): 1932–8. doi: 10.1016/j.pain.2012.06.006 22805779

[85] Butterworth PA, Urquhart DM, Cicuttini FM, Menz HB, Strauss BJ, Proietto J, et al. Fat mass is a predictor of incident foot pain. Obesity. 2013; 21(9): E495–9. doi: 10.1002/oby.20393 23512967

[86] Barber Foss KD, Hornsby M, Edwards NM, Myer GD, Hewett TE. Is body composition associated with an increased risk of developing anterior knee pain in adolescent female athletes? The Physician and sportsmedicine. 2012; 40(1): 13–9. doi: 10.3810/psm.2012.02.1947 22508247 PMC3398745

[87] Shiri R, Solovieva S, Husgafvel-Pursiainen K, Telama R, Yang X, Viikari J, et al. The role of obesity and physical activity in non-specific and radiating low back pain: the Young Finns study. Seminars in arthritis and rheumatism. 2013; 42(6): 640–50. doi: 10.1016/j.semarthrit.2012.09.002 23270761

[88] Heuch I, Hagen K, Zwart JA. A comparison of anthropometric measures for assessing the association between body size and risk of chronic low back pain: The HUNT study. PLoS ONE. 2015; 10(10): no pagination. doi: 10.1371/journal.pone.0141268 26506618 PMC4623972

[89] Jin X, Ding C, Wang X, Antony B, Laslett LL, Blizzard L, et al. Longitudinal associations between adiposity and change in knee pain: Tasmanian older adult cohort study. Seminars in arthritis and rheumatism. 2016; 45(5): 564–9. doi: 10.1016/j.semarthrit.2015.10.006 26596913

[90] Walsh TP, Gill TK, Evans AM, Yaxley A, Shanahan EM, Hill CL. Association of Fat Mass and Adipokines with Foot Pain in a Community Cohort. Arthritis Care and Research. 2016; 68(4): 526–33. doi: 10.1002/acr.22719 26315271

[91] Batsis JA, Zbehlik AJ, Barre LK, Mackenzie TA, Bartels SJ. The impact of waist circumference on function and physical activity in older adults: longitudinal observational data from the osteoarthritis initiative. Nutrition journal. 2014; 13: 81. doi: 10.1186/1475-2891-13-81 25106459 PMC4267442

[92] Hussain SM, Urquhart DM, Wang Y, Shaw JE, Magliano DJ, Wluka AE, et al. Fat mass and fat distribution are associated with low back pain intensity and disability: Results from a cohort study. Arthritis Research and Therapy. 2017; 19 (1) (no pagination)(26). doi: 10.1186/s13075-017-1242-z 28183360 PMC5301404

[93] Dario AB, Loureiro Ferreira M, Refshauge K, Luque-Suarez A, Ordonana JR, Ferreira PH. Obesity does not increase the risk of chronic low back pain when genetics are considered. A prospective study of Spanish adult twins. Spine Journal. 2017; 17(2): 282–90. doi: 10.1007/s12020-017-1326-1 27751965

[94] Pan F, Laslett L, Blizzard L, Cicuttini F, Winzenberg T, Ding C, et al. Associations Between Fat Mass and Multisite Pain: A Five-Year Longitudinal Study. Arthritis care & research. 2017; 69(4): 509–16. doi: 10.1002/acr.22963 27390162

[95] Hashimoto Y, Matsudaira K, Sawada SS, Gando Y, Kawakami R, Kinugawa C, et al. Obesity and low back pain: a retrospective cohort study of Japanese males. Journal of physical therapy science. 2017; 29(6): 978–83. doi: 10.1589/jpts.29.978 28626304 PMC5468219

[96] Shiri R, Falah-Hassani K, Heliovaara M, Solovieva S, Amiri S, Lallukka T, et al. Risk Factors for Low Back Pain: A Population-Based Longitudinal Study. Arthritis care & research. 2019; 71(2): 290–9. doi: 10.1002/acr.23710 30044543

[97] Brady SRE, Urquhart DM, Hussain SM, Teichtahl A, Wang Y, Wluka AE, et al. High baseline fat mass, but not lean tissue mass, is associated with high intensity low back pain and disability in community-based adults. Arthritis Research and Therapy. 2019; 21 (1) (no pagination)(165). doi: 10.1186/s13075-019-1953-4 31277706 PMC6612201

[98] Laslett LL, Menz HB, Otahal P, Pan F, Cicuttini FM, Jones G. Factors associated with prevalent and incident foot pain: data from the Tasmanian Older Adult Cohort Study. Maturitas. 2018; 118: 38–43. doi: 10.1016/j.maturitas.2018.10.004 30415753

[99] Walsh TP, Quinn SJ, Evans AM, Yaxley A, Chisholm JA, Kow L, et al. Fat mass, but not fat-free mass, predicts increased foot pain with obesity, independent of bariatric surgery. Surgery for Obesity & Related Diseases. 2018; 14(9): 1389–95. 30057094 10.1016/j.soard.2018.06.015

[100] Muthuri S, Cooper R, Kuh D, Hardy R. Do the associations of body mass index and waist circumference with back pain change as people age? 32 years of follow-up in a British birth cohort. BMJ Open. 2020; 10(12): e039197. doi: 10.1136/bmjopen-2020-039197 33310796 PMC7735102

[101] Pan F, Tian J, Cicuttini F, Jones G. Metabolic syndrome and trajectory of knee pain in older adults. Osteoarthritis Cartilage. 2020; 28(1): 45–52. doi: 10.1016/j.joca.2019.05.030 31394191

[102] Fearon A, Stephens S, Cook J, Smith P, Neeman T, Cormick W, et al. The relationship of femoral neck shaft angle and adiposity to greater trochanteric pain syndrome in women. A case control morphology and anthropometric study. Br J Sports Med. 2012; 46(12): 888–92. doi: 10.1136/bjsports-2011-090744 22547561 PMC3597182

[103] Celan D, Turk Z. The impact of anthropometric parameters on the incidence of low back pain. Collegium antropologicum. 2005; 29(1): 101–5. 16117306

[104] Yip YB, Ho SC, Chan SG. Tall stature, overweight and the prevalence of low back pain in Chinese middle-aged women. International journal of obesity and related metabolic disorders: journal of the International Association for the Study of Obesity. 2001; 25(6): 887–92. 10.1038/sj.ijo.0801557 11439304

[105] Dario AB, Ferreira ML, Refshauge K, Sanchez-Romera JF, Luque-Suarez A, Hopper JL, et al. Are obesity and body fat distribution associated with low back pain in women? A population-based study of 1128 Spanish twins. European Spine Journal. 2016; 25(4): 1188–95. doi: 10.1007/s00586-015-4055-2 26084786

[106] Walsh TP, Arnold JB, Gill TK, Evans AM, Yaxley A, Hill CL, et al. Foot pain severity is associated with the ratio of visceral to subcutaneous fat mass, fat-mass index and depression in women. Rheumatology International. 2017; 37(7): 1175–82. doi: 10.1007/s00296-017-3743-0 28516238

[107] Sakai Y, Matsui H, Ito S, Hida T, Ito K, Koshimizu H, et al. Sarcopenia in elderly patients with chronic low back pain. Osteoporosis and Sarcopenia. 2017; 3(4): 195–200. doi: 10.1016/j.afos.2017.09.001 30775530 PMC6372819

[108] Spyropoulos P, Chronopoulos E, Papathanasiou G, Georgoudis G, Koutis C, Kompoti A. Chronic low back pain and function of Greek office workers.2008. 129–35 p.

[109] Sutbeyaz ST, Sezer N, Koseoglu BF, Ibrahimoglu F, Tekin D. Influence of knee osteoarthritis on exercise capacity and quality of life in obese adults. Obesity. 2007; 15(8): 2071–6. doi: 10.1038/oby.2007.246 17712125

[110] Li H, George DM, Jaarsma RL, Mao X. Metabolic syndrome and components exacerbate osteoarthritis symptoms of pain, depression and reduced knee function. Ann Transl Med. 2016; 4(7): 133. doi: 10.21037/atm.2016.03.48 27162783 PMC4842398

[111] Hultman G, Nordin M, Saraste H, Ohlsèn H. Body composition, endurance, strength, cross-sectional area, and density of MM erector spinae in men with and without low back pain. J Spinal Disord. 1993; 6(2): 114–23. 8504222

[112] Briggs MS, Givens DL, Schmitt LC, Taylor CA. Relations of C-reactive protein and obesity to the prevalence and the odds of reporting low back pain. Arch Phys Med Rehabil. 2013; 94(4): 745–52. doi: 10.1016/j.apmr.2012.11.026 23187041

[113] Han TS, Schouten JS, Lean ME, Seidell JC. The prevalence of low back pain and associations with body fatness, fat distribution and height. International journal of obesity and related metabolic disorders: journal of the International Association for the Study of Obesity. 1997; 21(7): 600–7. doi: 10.1038/sj.ijo.0800448 9226492

[114] Bihari V, Kesavachandran C, Pangtey BS, Srivastava AK, Mathur N. Musculoskeletal pain and its associated risk factors in residents of National Capital Region. Indian journal of occupational and environmental medicine. 2011; 15(2): 59–63. doi: 10.4103/0019-5278.90375 22223951 PMC3249791

[115] Hodselmans AP, Dijkstra PU, Geertzen JH, van der Schans CP. Nonspecific chronic low back pain patients are deconditioned and have an increased body fat percentage. International journal of rehabilitation research Internationale Zeitschrift fur Rehabilitationsforschung Revue internationale de recherches de readaptation. 2010; 33(3): 268–70. doi: 10.1097/MRR.0b013e328335213f 20101188

[116] Ojoawo A, Oloagun aM, Bamiwoyec bS. Relationship Between Pain Intensity and Anthropometric Indices in Women with low back pain –A Cross-Sectional Study. J Phys Ther. 2011; 3(2): 45–51.

[117] Perry M, Straker L, O’Sullivan P, Smith A, Hands B. Fitness, motor competence, and body composition are weakly associated with adolescent back pain. The Journal of orthopaedic and sports physical therapy. 2009; 39(6): 439–49. doi: 10.2519/jospt.2009.3011 19487825

[118] Toda Y, Segal N, Toda T, Morimoto T, Ogawa R. Lean body mass and body fat distribution in participants with chronic low back pain. Archives of internal medicine. 2000; 160(21): 3265–9. doi: 10.1001/archinte.160.21.3265 11088088

[119] Tanamas SK, Wluka AE, Berry P, Menz HB, Strauss BJ, Davies-Tuck M, et al. Relationship between obesity and foot pain and its association with fat mass, fat distribution, and muscle mass. Arthritis Care Res (Hoboken). 2012; 64(2): 262–8. 21972207 10.1002/acr.20663

[120] Ozer Kaya D, Duzgun I, Baltaci G. Differences in body fat mass, muscular endurance, coordination and proprioception in woman with and without knee pain: a cross-sectional study. Acta orthopaedica et traumatologica turcica. 2014; 48(1): 43–9. doi: 10.3944/AOTT.2014.3135 24643099

[121] Iizuka Y, Iizuka H, Mieda T, Tajika T, Yamamoto A, Ohsawa T, et al. Association between neck and shoulder pain, back pain, low back pain and body composition parameters among the Japanese general population. BMC Musculoskeletal Disorders. 2015; 16: 333. doi: 10.1186/s12891-015-0759-z 26537689 PMC4634147

[122] Brooks C, Siegler JC, Marshall PW. Relative abdominal adiposity is associated with chronic low back pain: a preliminary explorative study. BMC public health. 2016; 16: 700. doi: 10.1186/s12889-016-3357-6 27485214 PMC4971654

[123] Frilander H, Solovieva S, Mutanen P, Pihlajamaki H, Heliovaara M, Viikari-Juntura E. Role of overweight and obesity in low back disorders among men: A longitudinal study with a life course approach. BMJ Open. 2015; 5(8): no pagination. doi: 10.1136/bmjopen-2015-007805 26297359 PMC4550727

[124] Frilander H, Viikari-Juntura E, Heliövaara M, Mutanen P, Mattila VM, Solovieva S, et al. Obesity in early adulthood predicts knee pain and walking difficulties among men: A life course study. European Journal of Pain. 2016; 20(8): 1278–87. doi: 10.1002/ejp.852 26996726

[125] Lee JY, Han K, McAlindon TE, Park YG, Park SH. Lower leg muscle mass relates to knee pain in patients with knee osteoarthritis. International journal of rheumatic diseases. 2016. doi: 10.1111/1756-185X.12896 27306837

[126] Butterworth PA, Menz HB, Urquhart DM, Cicuttini FM, Landorf KB, Pasco JA, et al. Fat Mass Is Associated with Foot Pain in Men: The Geelong Osteoporosis Study. Journal of Rheumatology. 2016; 43(1): 138–43. doi: 10.3899/jrheum.141331 26628606

[127] Shiri R, Solovieva S, Husgafvel-Pursiainen K, Taimela S, Saarikoski LA, Huupponen R, et al. The association between obesity and the prevalence of low back pain in young adults: the Cardiovascular Risk in Young Finns Study. Am J Epidemiol. 2008; 167(9): 1110–9. doi: 10.1093/aje/kwn007 18334501

[128] Alfieri FM, Silva N, Battistella LR. Study of the relation between body weight and functional limitations and pain in patients with knee osteoarthritis. Einstein. 2017; 15(3): 307–12. doi: 10.1590/S1679-45082017AO4082 29091152 PMC5823044

[129] Scott D, Blizzard L, Fell J, Jones G. Prospective study of self-reported pain, radiographic osteoarthritis, sarcopenia progression, and falls risk in community-dwelling older adults. Arthritis Care Res (Hoboken). 2012; 64(1): 30–7. 21739619 10.1002/acr.20545

[130] Machado LAC, Viana JU, Da Silva SLA, Couto FGP, Mendes LP, Ferreira PH, et al. Correlates of a recent history of disabling low back pain in community-dwelling older persons the pain in the elderly (PAINEL) study. Clin J Pain. 2018; 34(6): 515–24. doi: 10.1097/AJP.0000000000000564 29077624

[131] Endo T, Abe T, Akai K, Kijima T, Takeda M, Yamasaki M, et al. Height loss but not body composition is related to low back pain in community-dwelling elderlies: Shimane CoHRE study. BMC Musculoskeletal Disorders. 2019; 20(1): 207. doi: 10.1186/s12891-019-2580-6 31077175 PMC6511157

[132] Muramoto A, Imagama S, Ito Z, Hirano K, Tauchi R, Ishiguro N, et al. Waist circumference is associated with locomotive syndrome in elderly females. J Orthop Sci. 2014; 19(4): 612–9. doi: 10.1007/s00776-014-0559-6 24668310

[133] Ogwumike OO, Adeniyi AF, Orogbemi OO. Musculoskeletal pain among postmenopausal women in Nigeria: Association with overall and central obesity. Hong Kong Physiother J. 2016; 34: 41–6. doi: 10.1016/j.hkpj.2015.06.001 30931026 PMC6385138

[134] Brady SRE, Mousa A, Naderpoor N, de Courten MPJ, Cicuttini F, de Courten B. Adipsin Concentrations Are Associated with Back Pain Independently of Adiposity in Overweight or Obese Adults. Front Physiol. 2018; 9: 93. doi: 10.3389/fphys.2018.00093 29483883 PMC5816231

[135] Kulandaivelan S, Ateef M, Singh V, Chaturvedi R, Joshi S. One year prevalence of low back pain and its correlates in Hisar urban population. Journal of Musculoskeletal Research. 2018; 21(2).

[136] Yoshimoto T, Ochiai H, Shirasawa T, Nagahama S, Uehara A, Sai S, et al. Sex differences in the association of metabolic syndrome with low back pain among middle-aged Japanese adults: a large-scale cross-sectional study. Biol Sex Differ. 2019; 10(1): 33. doi: 10.1186/s13293-019-0249-3 31277712 PMC6612171

[137] Nava-Bringas TI, López-Domínguez L, Macías-Hernández SI, Espinosa-Morales R, Chávez-Arias DD, Coronado-Zarco R. Asociación de la composición corporal total con la fuerza del tronco, el dolor y la discapacidad en pacientes con espondiloartrosis lumbar. Cir Cir. 2018; 86(5): 388–91. doi: 10.24875/CIRU.18000006 30226492

[138] Hussien H, Kamel E, Kamel R. Association between pain intensity and obesity in patients with chronic non-specific low back pain. Bioscience Research. 2019; 16(4): 3579–83.

[139] Despres JP. Is visceral obesity the cause of the metabolic syndrome? Annals of medicine. 2006; 38(1): 52–63. doi: 10.1080/07853890500383895 16448989

[140] Ness-Abramof R, Apovian CM. Waist circumference measurement in clinical practice. Nutrition in clinical practice: official publication of the American Society for Parenteral and Enteral Nutrition. 2008; 23(4): 397–404. doi: 10.1177/0884533608321700 18682591

[141] Cao H. Adipocytokines in obesity and metabolic disease. The Journal of endocrinology. 2014; 220(2): T47–59. doi: 10.1530/JOE-13-0339 24403378 PMC3887367

[142] Neogi T. The epidemiology and impact of pain in osteoarthritis. Osteoarthritis Cartilage. 2013; 21(9): 1145–53. doi: 10.1016/j.joca.2013.03.018 23973124 PMC3753584

[143] Hartvigsen J, Natvig B, Ferreira M. Is it all about a pain in the back? Best practice & research Clinical rheumatology. 2013; 27(5): 613–23. 24315143 10.1016/j.berh.2013.09.008

