# Supplementary material for: Is adiposity associated with back and lower limb pain? A systematic review
Source: PLoS One. 2021 Sep 14;16(9):e0256720. doi: 10.1371/journal.pone.0256720 (PMC8439494; doi:10.1371/journal.pone.0256720)
Supplement: S1 Text — (DOCX) [file pone.0256720.s002.docx]

Database(s): **Ovid MEDLINE(R) and Epub Ahead of Print, In-Process & Other Non-Indexed Citations, Daily and Versions(R)**

Search Strategy:

| **#** | **Searches** |
| --- | --- |
| 1 | Musculoskeletal Pain/ or Back Pain/ or Low Back Pain/ or Metatarsalgia/ or Osteoarthritis, Hip/ or Osteoarthritis, Knee/ or Osteoarthritis, Spine/ or Patellofemoral Pain Syndrome/ |
| 2 | (musculoskeletal pain or back pain or low back pain or midback pain or knee osteoarthrit* or hip osteoarthrit* or knee arthrit* or hip arthrit* or knee arthralgia or hip arthralgia or patellofemoral pain or backache or backpain or vertebrogenic pain or lumbago or footache or kneepain or footpain or legpain or lower limbpain or lumbalgesia or lumbar spine syndrome or lumbosacroiliac pain).mp. |
| 3 | (cruralgia or coxalgia or coccyalgia or coccygalgia or dorsalgia or gonalgia or lumbalgia or meralgia or metatarsalgia or patellalgia or podalgia or plantalgia or sacralgia or sacrocoxalgia or spondylalgia or spinalgia or talalgia or tarsalgia or tibialgia or calcaneodynia or calcodynia or coccygodynia or coccydynia or coccyodynia or coxodynia or dorsodynia or lumbodynia or pododynia or sacrodynia or spondylodynia).mp. |
| 4 | ((lower limb* or lower extremet*) adj (pain* or ache*)).mp. |
| 5 | ((painful or aching) adj (lower limb* or lower extremet*)).mp. |
| 6 | ((lumbar or sacral or sacroiliac* or sacro-iliac* or iliosacral or il#o-sacral or sacrococcygeal or sacro-coccygeal or tailbone or coccyx or coccygeal or iliac crest or iliolumbar or il#o-lumbar or piriformis or discogenic or thoracolumbar or lumbosacral or lumbopelvic or lumbo-ischial or lumbar-ischial) adj (pain* or ache*)).mp. |
| 7 | ((hip*1 or hipbone* or leg*1 or thigh* or femur or femoral or knee* or patella* or popliteus or popliteal or tibial or shin* or crural or calf or fibular or ankle or tarsus or tarsal or heel or calcaneus or calcaneal or feet or foot or forefoot or midfoot or hindfoot or plantar or toe*1 or hallux or acetabulofemoral or acetabulo-femoral or tibiofemoral or tibio-femoral or patellofemoral or patello-femoral or astragalocrural or astragalo-crural or talo-crural or talocrural or metatarsophalangeal or metatarso-phalangeal or intertarsal or inter-tarsal or tibiotarsal or tibio-tarsal or tibiotalar or tibio-talar or talocalcanea* or talo-calcanea* or subtalar or sub-talar or tibiofibular or tibio-fibular or talonavicular or talo-navicular or tarsometatarsal or tarso-metatarsal or achilles or trochanter*) adj (pain* or ache*)).mp. |
| 8 | ((hamstring or psoas or quadriceps or iliopsoas or metatarsal or intermetatarsal or talus or fibula or tibia or sesamoid or ischial) adj (pain* or ache*)).mp. |
| 9 | 1 or 2 or 3 or 4 or 5 or 6 or 7 or 8 |
| 10 | body fat distribution/ or adiposity/ or obesity, abdominal/ or Body Composition/ |
| 11 | adipose tissue/ or adipose tissue, beige/ or adipose tissue, brown/ or adipose tissue, white/ or abdominal fat/ or intra-abdominal fat/ or subcutaneous fat, abdominal/ or subcutaneous fat/ or adipocytes/ or adipocytes, white/ |
| 12 | (abdominal obesity or obese abdomen or body composition*).mp. |
| 13 | (adipos* or adipocyte*).mp. |
| 14 | (fat adj (patterning or mass or deposit* or content or accumulat* or muscle or tissue or volume* or percentage or distribut* or thickness or ratio?)).mp. |
| 15 | ((body or bodily or trunk or subcutaneous or sub-cutaneous or visceral or abdominal or android or gynoid) adj3 fat).mp. |
| 16 | ((android or gynoid) adj2 (accumulat* or percentage* or distribut* or ratio?)).mp. |
| 17 | ((trunk or subcutaneous or sub-cutaneous or visceral or android or gynoid) adj obesity).mp. |
| 18 | 10 or 11 or 12 or 13 or 14 or 15 or 16 or 17 |
| 19 | 9 and 18 |
| 20 | exp animals/ not humans.sh. |
| 21 | 19 not 20 |
| 22 | limit 21 to (case reports or editorial or letter or news) |
| 23 | 21 not 22 |
| 24 | (pregnan* or gestation* or antenatal or prenatal or natal or gravidit* or gravida* or multigravid* or primigravid*).mp. |
| 25 | 23 not 24 |
| 26 | (newborn* or neonat* or infant* or infancy or preschool* or pre-school*).mp. |
| 27 | 25 not 26 |
| 28 | limit 27 to ("newborn infant (birth to 1 month)" or "infant (1 to 23 months)" or "preschool child (2 to 5 years)" or "child (6 to 12 years)") |
| 29 | 27 not 28 |
| 30 | limit 29 to english language |
